# Supplementary material for: Roles of mothers and fathers in supporting child physical activity: a cross-sectional mixed-methods study
Source: BMJ Open. 2018 Jan 21;8(1):e019732. doi: 10.1136/bmjopen-2017-019732 (PMC5781024; doi:10.1136/bmjopen-2017-019732)
Supplement: Supplementary file 1 [file bmjopen-2017-019732supp001.pdf]

Table S1 Number of mothers and fathers who were eligible for and recruited to the interview study

No.	Sub-group description	N parents eligible in each sub-group Total	Mothers	Fathers	N parents recruited in each sub-group Total	Mothers	Fathers
1	Low MVPA, low SED boys	31	20	11	6	4	2
2	High MVPA, low SED boys	116	82	34	6	3	3
3	Low MVPA, high SED boys	67	51	16	7	5	2
4	High MVPA, high SED boys	63	48	15	6	3	3
5	Low MVPA, low SED girls	69	48	21	6	3	3
6	High MVPA, low SED girls	86	67	19	6	4	2
7	Low MVPA, high SED girls	138	111	27	7	4	3
8	High MVPA, high SED girls	55	37	18	7	5	2
	**Total**	**625**	**464**	**161**	**51**	**31**	**20**

Table S2 Frequency of reporting which parent supports child activity by parent and separately by child gender

		Parent gender			Child gender		
		**Males (N=264) %**	**Females (N=680) %**	**Chi-squared p-value for difference**	**Boys (N=427) %**	**Girls (N=517) %**	**Chi-squared p-value for difference**
**Supports child activity during the week**	Mother	26.9	57.4	<0.001	44.5	52.4	0.04
	Father	16.7	2.9		6.6	7.0	
	Both parents	56.4	39.7		48.9	40.6	
**Supports child activity at the weekend**	Mother	8.3	30.7	<0.001	21.1	27.3	0.02
	Father	26.9	14.1		20.8	15.1	
	Both parents	64.8	55.1		58.1	57.6	
**Who should support child activity**	Mother	1.1	6.9	0.001	4.2	6.0	0.07
	Father	1.5	0.7		1.6	0.4	
	Both parents	97.3	92.4		94.1	93.6	
