# Supplementary material for: Roles of mothers and fathers in supporting child physical activity: a cross-sectional mixed-methods study
Source: BMJ Open. 2018 Jan 21;8(1):e019732. doi: 10.1136/bmjopen-2017-019732 (PMC5781024; doi:10.1136/bmjopen-2017-019732)
Supplement: Author's manuscript [file bmjopen-2017-019732.draft_revisions.pdf]

BMJ Open is committed to open peer review. As part of this commitment we make the peer review history of every article we publish publicly available.

When an article is published we post the peer reviewers' comments and the authors' responses online. We also post the versions of the paper that were used during peer review. These are the versions that the peer review comments apply to.

The versions of the paper that follow are the versions that were submitted during the peer review process. They are not the versions of record or the final published versions. They should not be cited or distributed as the published version of this manuscript.

BMJ Open is an open access journal and the full, final, typeset and author-corrected version of record of the manuscript is available on our site with no access controls, subscription charges or pay-per-view fees (http://bmjopen.bmj.com).

If you have any questions on BMJ Open's open peer review process please email editorial.bmjopen@bmj.com

BMJ Open

Who leads and who supports? A cross-sectional mixed methods study of mothers' and fathers' support for child physical activity

Journal:	*BMJ Open*
Manuscript ID	bmjopen-2017-019732
Article Type:	Research
Date Submitted by the Author:	21-Sep-2017
Complete List of Authors:	Solomon-Moore, Emma; Univ Bristol<br>Toumpakari, Zoi; University of Bristol, Centre for Exercise, Nutrition and Health Sciences<br>Sebire, Simon; University of Bristol<br>Thompson, Janice; The University of Birmingham, School of Sport & Exercise Sciences<br>Lawlor, Debbie; Department of Social Medicine, University of Bristol, MRC Integrative Epidemiology Unit<br>Jago, Russ; University of Bristol, Centre for Exercise, Nutrition and Health Sciences
**Primary Subject Heading**:	Public health
Secondary Subject Heading:	Paediatrics
Keywords:	Physical activity, children, parents, gender, mixed-methods

SCHOLARONE™
Manuscripts

For peer review only - http://bmjopen.bmj.com/site/about/guidelines.xhtml

Who leads and who supports? A cross-sectional mixed methods study of mothers' and fathers' support for child physical activity

Emma Solomon-Moore [1], Zoi Toumpakari [1], Simon J. Sebire [1], Janice L. Thompson [2], Deborah A. Lawlor [3, 4], and Russell Jago [1$]

[1] Centre for Exercise, Nutrition & Health Sciences, School for Policy Studies, University of Bristol, 8 Priory Road, Bristol, BS8 1TZ, UK.

[2] School of Sport, Exercise and Rehabilitation Sciences, University of Birmingham, Birmingham, B15 2TT, UK.

[3] MRC Integrative Epidemiology Unit at the University of Bristol, Oakfield House, Oakfield Grove, Bristol, BS8 2BN, UK.

[4] Population Health Science, Bristol Medical School, University of Bristol, Canynge Hall, Whiteladies Road, Bristol, BS8 2PS, UK.

[$] **Corresponding author:** Professor Russell Jago, Centre for Exercise, Nutrition & Health Sciences, School for Policy Studies, University of Bristol, 8 Priory Road, Bristol, BS8 1TZ, UK. Email: russ.jago@bristol.ac.uk; Telephone: +44 (0)117 9546603

Email addresses

ESM: Emma.solomon@bristol.ac.uk

ZT: Z.Toumpakari@bristol.ac.uk

SJS: simon.sebire@bristol.ac.uk

JLT: j.thompson.1@bham.ac.uk

DAL: d.a.lawlor@bristol.ac.uk

RJ: russ.jago@bristol.ac.uk

Word count: **Manuscript** (excluding tables) = 4480 words **Abstract** = 300 words

For peer review only - http://bmjopen.bmj.com/site/about/guidelines.xhtml

ABSTRACT

Objectives: Examine the extent to which parent gender is associated with supporting children's physical activity.

Design: Cross-sectional mixed-methods study.

Setting: 47 primary schools located in Bristol (UK).

Participants: 944 8-9-year-old children and one of their parents provided quantitative data; 51 parents were interviewed.

Methods: Children wore an accelerometer and mean minutes of moderate-to-vigorous-intensity physical activity (MVPA) per day, counts per minute (CPM), and achievement of national MVPA guidelines were derived. Parents reported who leads in supporting child activity during the week and weekend. Linear and logistic regression examined the association between gender of parent who leads child activity and child physical activity. For the semi-structured telephone interviews, inductive and deductive content analysis were used to explore the role of gender in how parents lead child activity.

Results: Parents appeared to have a stronger role in supporting boys to be more active, than girls, and the strongest associations were when they reported that both parents had equal roles in supporting their child. For example, compared with the reference of female/mother leading support, equal contribution from both parents was associated with boys doing 5.9 (95% CI: 1.2 to 10.6) more minutes of MVPA per day during the week, and more CPM on both week and weekend days (55.1 [14.3 to 95.9] and 52.8 [1.8 to 103.7], respectively). Associations in girls were weaker and sometimes in the opposite direction but there was no strong statistical evidence for gender interactions. Qualitatively, parents described a range of support arrangements, commonly; proactively supporting physical activity equally, mothers leading

For peer review only - http://bmjopen.bmj.com/site/about/guidelines.xhtml

during the week, families getting together at weekends, families doing activities separately due to preferences, and parents using activities to bond one-to-one with children.

Conclusions: Mothers primarily lead child activity during the week. Children, possibly more so boys, are more active if both parents share the supporting role.

Key words: Physical activity, children, parents, gender, mixed-methods

For peer review only

ARTICLE SUMMARY

Strengths and limitations of this study

Strengths

- Mixed-methods study.
- Accelerometer data from a large sample of 8-9-year-old children.
- Semi-structured interviews with 51 parents, including 20 fathers.

Limitations

- Cross-sectional study design from a single UK region.
- The measurement of parental leadership of child physical activity would be strengthened by collecting data from both parents and information on the quality and quantity of leadership.

For peer review only - http://bmjopen.bmj.com/site/about/guidelines.xhtml

INTRODUCTION

Children who are physically active are at a lower risk of obesity, high blood pressure, metabolic syndrome, and depression.[1 2] The UK Government recommends that children and young people aged 5 to 18 years should engage in at least 60 minutes of moderate-to-vigorous-intensity physical activity (MVPA) every day.[3] However, data from the nationally representative Millennium cohort showed that only 51% of 7-8 year olds met the recommendation.[4] Physical activity declines throughout childhood and adolescence, with boys being more active than girls at all ages.[4-9] Thus, in order to develop effective means of increasing child physical activity, there is a need to understand the factors that influence behaviour.

Parents act as gatekeepers to children's activity,[10] and can play an important role in increasing their child's physical activity.[11-13] For instance, parents can influence their child's activity by being active with their child, role-modelling active behaviour, and/or by facilitating physical activity for their child (logistic support).[13-16] Studies examining links between parent and child physical activity have yielded mixed results.[14 17-20] A growing body of work has shown that providing logistic support is associated with increased physical activity,[21-23] and therefore, may be the most important source of parental influence on children's activity.

The gender of the parent who takes the lead in supporting child activity could be an important influence on children's activity levels. Several studies suggest that mothers play a larger role in the logistical planning of children's physical activity, while fathers are more likely to model physical activity.[24 25] However, most studies in this area have focused on the

For peer review only - http://bmjopen.bmj.com/site/about/guidelines.xhtml

mother-child relationship, and relatively little attention has been paid to the role of fathers.[26] From qualitative interviews with parents of 5-6-year-old children in the B-Proact1v study, we found evidence that fathers play a key role in promoting children's physical activity, influencing their choices and behaviours,[27] a finding replicated in other studies.[28 29] The Healthy Dads, Healthy Kids intervention demonstrated that engaging fathers in physical activity with their children can promote increases in children's physical activity.[30 31] Data from the B-Proact1v interviews suggest that fathers may take more responsibility for their son's physical activity (e.g., taking their son to sports clubs), and mothers with their daughter's activity.[27] To date, there is inconsistent evidence regarding whether gender-specific parental influence (i.e., mothers with daughters and fathers with sons) is stronger than cross-gender parental influence (i.e., mothers with sons and fathers with daughters) on children's physical activity.[24 32-35] Therefore, a greater understanding is needed about the role gender plays in how parents support their child to be active, and if this varies by child gender.

The aim of this mixed-methods study was to examine parent gender, in terms of which parent takes the lead in supporting their child to be active, and its association with child physical activity. A secondary aim was to discover if these associations varied by child gender.

METHODS

Data are from the longitudinal B-Proact1v study, which aimed to examine factors associated with children's and parents' physical activity and screen-viewing behaviours. The study has been described in detail elsewhere.[9 17 36] Briefly, in 2012 and 2013, data were collected from 1299 Year 1 children (5-6 years old) from 57 primary schools across Bristol, UK.

For peer review only - http://bmjopen.bmj.com/site/about/guidelines.xhtml

Between March 2015 and July 2016, 47 of the original schools were re-recruited and data were collected from 1223 Year 4 children (8-9 years old). One of the children's parents were also recruited to the study. The current study used cross-sectional data from the Year 4 assessments, for the 944 children and parents who provided valid child accelerometer data and complete parent questionnaire data for questions on child and parent demographics and gender roles associated with supporting child activity (Figure 1). In addition, we drew on qualitative data via semi-structured telephone interviews from a sub-sample of 51 parents (details below; Figure 2). The study received ethical approval from the School for Policy Studies Ethics Committee at the University of Bristol, and written parent consent was received for all participants.

Accelerometer data

Children wore a waist-worn ActiGraph wGT3X-BT accelerometer for five days including two weekend days. Accelerometer data were processed using Kinesoft (v3.3.75; Kinesoft, Saskatchewan, Canada), and were included in the primary analyses if children provided at least three days of valid data (including at least one weekend day). A valid day was defined as at least 500 minutes of data after excluding intervals of ≥60 minutes of zero counts, allowing up to two minutes of interruptions. Minutes spent in MVPA were derived using population-specific cut points for children.[37] Mean accelerometer counts per minute (CPM), and a binary variable indicating whether the child's average daily MVPA was greater than the 60 minutes per day recommended by the UK government,[3] were also derived.

Parent leadership variables

For peer review only - http://bmjopen.bmj.com/site/about/guidelines.xhtml

To understand the gender roles associated with parents supporting their child's activity, parents were asked three questions via a questionnaire: a) "In your family who takes the lead role in supporting your Year 4 child to be active during the week?", b) "In your family who takes the lead role in supporting your Year 4 child to be active at the weekend?" and c) "Who do you think should take the lead role in supporting your Year 4 child to be active?". Each question had three response options: "Mother/Female care-giver", "Father/Male care-giver" or "About the same" for questions a) and b), and "Should be shared" for question c).

Demographic information

Parents provided demographic information via a questionnaire, including parent and child gender and date of birth. Where children's date of birth was missing (21% of children) they were assigned the median age of 9.0 years (as the children were all in the same school year with a maximum age difference between the youngest and oldest of just under 12-months legally possible). Indices of Multiple Deprivation (IMD) scores, based upon the English Indices of Deprivation (http://data.gov.uk/dataset/index-of-multiple-deprivation), were assigned to each child based on their reported home postcode.

Interview data

During consent procedures, parents were informed that they may be re-contacted to take part in a telephone interview. Only families with complete data for all measures (child and parent accelerometer data, child height, weight and blood pressure, and child and parent questionnaire data) were included in the interview sample (N=625, of which 161 (25.8%) had data from fathers). This sample was stratified according to the child's MVPA minutes per day (dichotomised around the study median: 57.5 minutes), sedentary minutes per day

For peer review only - http://bmjopen.bmj.com/site/about/guidelines.xhtml

(dichotomised around the median: 434.6 minutes), and by child gender. This produced eight sub-groups (1 = low MVPA, low sedentary time boys; and 8 = high MVPA, high sedentary time girls; Table S1). The order in which parents were invited to participate in an interview was randomised within each sub-group. Contact attempts were made with 188 parents in total, of which 59 (31.4%) initially agreed to participate in an interview, and 51 (27.1%) completed an interview (Figure 2). Interviews were audio-recorded and continued until theoretical saturation was reached for the entire sample and the sub-groups. Parents were invited to participate by telephone between July and October 2016, and interviews were conducted at the interviewee's convenience (37 during weekday daytimes (72.5%), 13 during weekday evenings (25.5%), and 1 on a weekend evening (2%)). Participants were sent a £10 high street shopping voucher as a thank you for their time.

An interview guide was developed and refined by the research team based on identifying gaps in current knowledge and guided by the Year 1 B-Proact1v quantitative and qualitative findings. This included questions relating to a variety of topics, including parents' perceptions of their child's physical activity and screen-viewing behaviours, strategies for managing these behaviours, understanding what has changed regarding these behaviours, and parents' experiences from their own childhood. Questions were posed in a non-leading manner to allow participants to shape the direction of the interview, and issues that emerged were probed. Interviews were conducted by two female researchers (qualified to at least MSc level) who were trained in conducting qualitative interviews.

Mixed-methods approach

For peer review only

For peer review only - http://bmjopen.bmj.com/site/about/guidelines.xhtml

The current study uses a mixed-methods design, incorporating quantitative data from questionnaires and accelerometry, with qualitative data from semi-structured interviews. Combining quantitative and qualitative approaches can provide a better understanding of research problems than either approach alone,[38] and the opportunity to present a greater diversity of divergent views.[39] Mixed-methods research is not designed to replace either qualitative or quantitative research, but rather to extract the strengths and diminish the weaknesses in both approaches within a single study.[40] Although many designs exist, there are three primary mixed-methods models: 1) convergent parallel mixed-methods, in which quantitative and qualitative research are conducted at roughly the same time and then integrated to provide a comprehensive analysis of the research problem; 2) explanatory sequential mixed-methods – quantitative research is conducted and analysed and then qualitative research is introduced to build on the results and explain them in more detail; and 3) exploratory sequential mixed-methods – qualitative research is initially conducted and analysed to explore the views of participants, and this information is used to build into a second, quantitative phase.[41] The current study incorporated a convergent parallel mixed-methods design, although quantitative data were collected prior to qualitative data collection, analyses and interpretation were conducted in parallel.

Data analysis

Quantitative data

Means, proportions and Chi Square statistics were used to examine the distributions of exposures, outcomes and co-variates between participants included and excluded in this study, and between child and parent gender. Nearly all parents reported that both parents "*should take the lead*" in supporting their child's activity (93.8%), therefore we could not

For peer review only - http://bmjopen.bmj.com/site/about/guidelines.xhtml

explore the association of parental attitudes towards who should lead in supporting child physical activity, as numbers were too small in the mother or father only categories. We used linear regression models to examine the associations of parent leading child activity during the week and weekend with the child's MVPA minutes per day and CPM, and logistic regression models to examine associations with achievement of the MVPA guideline. Models were adjusted for child age, gender of parent providing the information on lead support, and household IMD score. Robust standard errors were used to account for the clustering of children in schools for all models. Models were examined for all children, and separately for boys and girls. Combined Wald tests were used to test for evidence of interaction between child gender and the exposure of interest. All analyses were performed in Stata version 14.0 (StataCorp, 2015).

Qualitative data

Interviews were transcribed verbatim and anonymised before being entered into QSR NVivo 10 (QSR International, Warrington UK) to facilitate analysis. Using the framework method, thematic content analysis was performed by two researchers, enabling themes to develop both inductively from the accounts (experiences and views) of participants and deductively from existing literature.[42,43] Analysis involved several phases: familiarisation, coding, developing a framework, applying the framework, charting data into the framework matrix, and interpretation. During familiarisation, transcripts were thoroughly read and re-read independently by two researchers to immerse themselves in the data. After discussion between the two researchers, an initial coding frame was developed and applied to the data based on pre-existing ideas, and was refined throughout the process to allow for the inductive emergence of additional themes. The two researchers met regularly to ensure accuracy and

For peer review only - http://bmjopen.bmj.com/site/about/guidelines.xhtml

consistency. Hierarchies of categories were created and summarised, and brief summaries, mind maps, and representative quotes for each category were abstracted for reporting purposes. The final quotes were selected as they are illustrative of several responses given by parents.

RESULTS

Participant characteristics

The characteristics of the participants included and excluded from the quantitative dataset, and from the subset of interview participants, are shown in Table 1. Of the 944 included families, the majority (680 (72%)) had data from a mother/female care giver, with 264 (28%) from fathers/male care givers. Children excluded due to missing data were more likely to be deprived and did less minutes of MVPA per day, but were otherwise similar to the included dataset. Of the interview participants (N=51), 31 were mothers and 20 were fathers, with an average age of 41.2 (SD: 4.5) years, and 94.1% were White British. The interview participants were generally comparable to the main dataset, but tended to be less deprived. Interview participants were also more likely to be fathers and have less active children compared to the main dataset. The average interview duration was 34.4 minutes (SD: 8.0 minutes, range: 18 to 55 minutes).

For peer review only - http://bmjopen.bmj.com/site/about/guidelines.xhtml

Characteristic	Included (N=944)	Excluded			Interview sample (N=51)
	Mean (SD) or %	N	Mean (SD) or %	*p*	Mean (SD) or %
Child MVPA (mins/day)	62.8 (22.8)	209	58.6 (21.4)	0.01	58.3 (17.4)
Accelerometer counts per minute	620.4 (203.2)	209	609.0 (208.8)	0.46	573.2 (142.0)
Met MVPA guidelines (≥60 mins/day)		209		0.06	
No	52.0		59.3		58.8
Yes	48.0		40.7		41.2
Child gender		279		0.73	
Boy	45.2		46.4		49.0
Girl	54.8		53.6		51.0
Age of child (years)	9.03 (0.46)	279	9.04 (0.49)	0.91	8.95 (0.37)
Household IMD[b] score	15.1 (13.6)	248	18.8 (15.5)	<0.001	11.5 (9.7)
Takes lead role in child activity during the week		39		0.92	
Mother	48.8		48.7		43.1
Father	6.8		5.1		9.8
Both parents	44.4		46.2		47.1
Takes lead role in child activity at the weekend		37		0.35	
Mother	24.5		32.4		23.5
Father	17.7		21.6		23.5
Both parents	57.8		45.9		52.9
Who should take lead role in child PA		38		0.64	
Mother	5.2		2.6		3.9
Father	1.0		0.0		3.9
Both parents	93.8		97.4		92.2
Parent gender		41		0.24	
Male	28.0		19.5		39.2
Female	72.0		80.5		60.8

For peer review only - http://bmjopen.bmj.com/site/about/guidelines.xhtml

276 **Table 1 Descriptive characteristics of the main study sample (N=944) and subset of interview participants (N=51)**

277 MVPA: Moderate-to-vigorous physical activity; IMD: Index of multiple deprivation; a higher value indicates greater deprivation

For peer review only

For peer review only - http://bmjopen.bmj.com/site/about/guidelines.xhtml

Supplementary Table 2 shows the gender of the parent who reportedly leads child physical activity by parent and child gender. Mothers reported that typically they led in supporting their child's physical activity during the week, whereas fathers generally reported that duties were shared between parents. Most mothers and fathers reported that both parents shared the role of supporting their child's activity at the weekend, however, 31% of mothers and 27% of fathers, respectively, reported that they led child activity.

The interview data generally supported this, with several mothers stating that they took the lead in supporting their child to be active during the week out of necessity because fathers were working long hours or late into the evening. Some mothers also reported that they try to get the whole family together to do activities at the weekend, although this isn't always the norm.

"On a weekday it's just, you know, every night we've got one or the other [children] have got a club on so it's just finish school and then me taking the children to their various clubs and then coming home and it's, erm, you know, pretty much get ready for bedtime ... Weekends, yeah, we try to do stuff as a family." [Int 14, Mother, Girl, 63 MVPA minutes/day, Mother leads weekday PA, Both parents lead weekend PA]

"We like to do things as a family when we can; it's just all being around. My husband works quite late hours and things like that ... He's, he's home when they're going to bed usually ... but like last Sunday, we all went swimming together as a family thing... but that isn't – to be honest, that isn't like, isn't like we would do that every weekend or anything" [Int 35,

For peer review only

For peer review only - http://bmjopen.bmj.com/site/about/guidelines.xhtml

Mother, Girl, 72 MVPA minutes/day, Mother leads weekday PA, Both parents lead weekend PA]

Some parents indicated that they share the responsibility of leading child physical activity, due to sharing an appreciation for the benefits of physical activity or because they value physical activity and feel a moral responsibility to fit activity in to the realities of life.

"I'm active, my husband's active. And so, you know, we cascade that if you like down to the children so we, we don't really sit around at all, we're very active and on the go…" [Int 3, Mother, Son, 59 MVPA minutes/day, Both parents lead weekday and weekend PA]

"Actively we are trying to get the children involved in the various, activities like where there's after-school or a swimming lesson or they are going to join Scouts, which will be helpful for them in the long run… So, so we, we are encouraging them to get involved in outdoor activities as much as possible." [Int 1, Father, Son, 76 MVPA minutes/day, Both parents lead weekday and weekend PA]

"So wherever we can we'll always try and do the right thing and, you know, sometimes if it's not taking the car and it's walking distance we'll try and walk, and things like that.." [Int 18, Father, Son, 86 MVPA minutes/day, Father leads weekday and weekend PA]

A few parents reported sharing the responsibility of leading child physical activity, but also doing activities separately due to child preferences. Examples included fathers and sons using physical activity time to bond over shared interests, while also giving mothers a break for

For peer review only - http://bmjopen.bmj.com/site/about/guidelines.xhtml

some "me time", or parents taking children to separate activities to appease child preferences, avoid conflict, and/or facilitate parent-child one-on-one time irrespective of gender.

"We like going about walking as a family. Well, I say me and my husband do and we drag the kids along, but, you know, it's just getting some fresh air, but the boys have their own interests as well, such as the rugby or football which my husband takes the boys to. I have a bit of 'me time' when they go off to do that so, you know, it's a mix, I think." [Int 32, Mother, Girl, 86 MVPA minutes/day, Both parents lead weekday and weekend PA]

"I would like to do a little bit more with them but because my son doesn't like what [child] likes and I would like to take them swimming together a little bit more so we can all go and do swimming but because he doesn't like it; we kind of end up two of us doing it and two of us not doing it" [Int 29, Mother, Girl, 56 MVPA minutes/day, Both parents lead weekday and weekend PA]

"I've said I might take him mountain biking this Sunday because I see that as exercise for him but also one to one. So, he's getting that, the benefit of obviously exercise, the sport that he actually really loves and is getting one to one time with a parent where, you know, it's hard isn't it, when there's other siblings" [Int 3, Mother, Son, 59 MVPA minutes/day, Both parents lead weekday and weekend PA]

In the quantitative dataset, parents of girls tended to report that mothers take the lead in supporting their daughter's activity during the week, while parents of boys tended to report that the role was shared between both parents. Parents of boys and girls generally reported

For peer review only - http://bmjopen.bmj.com/site/about/guidelines.xhtml

that they shared the responsibility of leading child activity at the weekend, although parents of girls were more likely to report that mothers lead in supporting their daughter's weekend activity.

In contrast, the interview data revealed a mix of gender patterns associated with supporting child physical activity, not just mothers supporting daughters and fathers supporting sons. Some fathers reported they lead in supporting their daughter's physical activity through chauffeuring them to sports clubs, and expressed that they do so not just for logistical reasons, but also because they get real enjoyment from watching. A few mothers reported a lack of confidence in their own physical activity, because they aren't "naturally sporty" and so they tend to let fathers take the lead in supporting child physical activity.

"Yeah, she's, she's been - she's been playing football for err two and a half seasons now … so that's - and she's passionate about that. So I'm just a sort of chauffeur dad … that stands on the touchline in the cold windy rain. I, I enjoy that." [Int 51, Father, Girl, 71 MVPA minutes/day, Father leads weekday and weekend PA]

"Not that confident cause, like I say, I'm not actually naturally sporty or active. So it would be something that we would probably do as a family with their dad, and we could do it together……He's more confident, yeah, and he's more knowledgeable really with all that kind of stuff. And he's a – and he's the kind of person that's very much into, 'Come on, let's give it a go. Let's try and see. We might really enjoy it,' whereas I'm a bit more like, 'Oh no, don't make me do this. I'm really nervous.' And so I would probably shy away from it." [Int

For peer review only - http://bmjopen.bmj.com/site/about/guidelines.xhtml

24, Mother, Girl, 43 MVPA minutes/day, Mother leads weekday PA, Father leads weekend PA]

Associations of who leads child activity with child physical activity variables

Table 2 shows the mean difference in child MVPA minutes per day by which parent/s take the lead in supporting child activity during the week and weekend. Compared to reporting that mothers lead child activity (reference group), reporting that parents share the role of supporting child activity during the week was associated with children doing, on average, an additional 3.5 minutes of MVPA per day. When examined separately by child gender, parents sharing the role of leading child activity during the week was associated with, on average, an additional 5.9 minutes of MVPA per day for boys, and 0.4 minutes per day for girls, with no strong statistical evidence of a difference between boys and girls ($P_{interaction} = 0.34$). Fathers taking the lead in supporting child activity (compared to mothers) was more weakly associated with child MVPA, with an inverse (rather than positive) association for girls, but again with no strong statistical evidence for gender interaction. Associations for parent leadership of child physical activity during the weekend showed very similar patterns to those for weekday activity, but were somewhat weaker in magnitude. In general, the patterns of association with achieving MVPA recommendations were similar to what was found for MVPA as a continuous measure, including point estimates suggesting weaker or inverse effects in girls but no evidence of gender interaction (Table 3). The one exception was that fathers supporting activity at weekends had a similar magnitude of effect as both parents being lead supporters.

For peer review only - http://bmjopen.bmj.com/site/about/guidelines.xhtml

The mean difference in children's CPM by parent/s who lead child activity during the week also showed a similar pattern to that seen for time spent in MVPA (Table 2).

For peer review only - http://bmjopen.bmj.com/site/about/guidelines.xhtml

Table 2 Mean difference in the children's average MVPA minutes per day and accelerometer counts per minute associated with gender of parent who leads physical activity during the week and weekend (N=944)

Exposure		Moderate-to-vigorous physical activity (minutes/day): mean difference (95% confidence interval)			P for gender interaction
		All (N=944)	Boys (N=427)	Girls (N=517)	
**Takes leads role in child activity during week**	Mother (ref)	0	0	0	0.34
	Father	0.3 (-5.7, 6.3)	8.1 (-1.7, 17.9)	-3.7 (-10.4, 2.9)	
	Both parents	3.5 (0.6, 6.5)	5.9 (1.2, 10.6)	0.4 (-3.0, 3.8)	
**Takes lead role in child activity at the weekend**	Mother (ref)	0	0	0	0.22
	Father	1.7 (-2.8, 6.2)	5.7 (-1.5, 12.9)	-3.4 (-8.5, 1.7)	
	Both parents	2.4 (-1.1, 5.9)	4.5 (-1.4, 10.3)	0.7 (-3.0, 4.4)	
**Exposure**		**Accelerometer counts per minute: mean difference (95% confidence interval)**			
		**All (N=944)**	**Boys (N=427)**	**Girls (N=517)**	**P for gender interaction**
**Takes leads role in child activity during week**	Mother (ref)	0	0	0	0.61
	Father	0.7 (-51.7, 53.2)	56.7 (-28.8, 142.1)	-22.8 (-86.7, 41.1)	
	Both parents	28.0 (2.0, 54.0)	55.1 (14.3, 95.9)	2.8 (-29.9, 35.4)	
**Takes lead role in child activity at the weekend**	Mother (ref)	0	0	0	0.33
	Father	13.1 (-26.5, 52.6)	55.6 (-7.2, 118.3)	-26.2 (-75.9, 23.4)	
	Both parents	22.6 (-7.7, 52.9)	52.8 (1.8, 103.7)	4.7 (-31.3, 40.7)	

For peer review only

For peer review only - http://bmjopen.bmj.com/site/about/guidelines.xhtml

MVPA: Moderate-to-vigorous physical activity; Models are adjusted for child age, parent gender and household IMD score

Table 3 Odds ratio for children achieving 60 minutes of MVPA per day associated with gender of parent leading physical activity during the week and weekend (N=944)

Exposure		Meeting government guideline: odds ratio (95% confidence interval)			P for gender interaction
		All (N=944)	Boys (N=427)	Girls (N=517)	
**Takes leads role in child activity during week**	Mother (ref)	0	0	0	0.95
	Father	0.96 (0.54, 1.72)	1.61 (0.62, 4.21)	0.75 (0.34, 1.66)	
	Both parents	1.60 (1.20, 2.14)	2.23 (1.37, 3.62)	1.23 (0.83, 1.82)	
**Takes lead role in child activity at the weekend**	Mother (ref)	0	0	0	0.30
	Father	1.20 (0.78, 1.86)	2.10 (1.02, 4.32)	0.74 (0.40, 1.38)	
	Both parents	1.20 (0.86, 1.68)	1.81 (1.01, 3.24)	1.00 (0.64, 1.54)	

For peer review only - http://bmjopen.bmj.com/site/about/guidelines.xhtml

MVPA: Moderate-to-vigorous physical activity; Models are adjusted for child age, parent gender and household IMD score

For peer review only

For peer review only - http://bmjopen.bmj.com/site/about/guidelines.xhtml

DISCUSSION

The data presented in this paper show that while parents believe the responsibility of leading child physical activity should be shared between both of them, quantitative data suggest that families mostly share the role on the weekend, with mothers primarily leading child activity during the week. This finding was mirrored in the interview data, where several mothers reported that they led child activity during the week, because fathers worked long hours or late into the evening. Traditional familial roles are shifting, and it is now more common for both parents to work and for fathers to take on the role of primary care provider,[44 45] so it may be expected that more fathers are taking an active role in their children's physical activity. We found that the majority of parents reported they shared the leadership role for supporting their child's activity both during the week and at the weekend (40-65% of mothers and fathers responded this way for both time points; Table S2).

In quantitative analyses for all three outcomes (time spent in MVPA, meeting MVPA recommendations and CPM) we saw similar patterns of, in general, higher child physical activity where parents reportedly shared the role of supporting their child's physical activity during both weekdays and weekends. The one exception was for meeting MVPA recommendations at the weekend, where associations of fathers reportedly taking the lead were similar to those when both parents shared the responsibility. There was some evidence that positive associations were stronger for sons, and that some associations were inverse for daughters. However, we found no strong statistical evidence that associations differed between sons and daughters, and without further exploration in much larger numbers we cannot assume that parental roles in supporting their child's activity differ by the child's gender.

For peer review only - http://bmjopen.bmj.com/site/about/guidelines.xhtml

There was some suggestion that mothers were more likely to lead in supporting their daughter to be active, while fathers were more likely to support their son's activity, though caution is needed here given the disparity in which parents provide data, with 72% of families having data from mothers only and 28% from fathers only. Several studies have reported that fathers may be more involved in their son's physical activity,[15 27] or have found stronger links between father-son and mother-daughter dyads in terms of their physical activity behaviour.[32-34] In contrast, interview data from the current study revealed a myriad of gender patterns, including examples from fathers supporting girls' physical activity because they were more confident than mothers at leading physical activity or because they enjoy watching their daughter play football, and a mother taking her son mountain biking to engage in quality one-on-one time. There were also examples of fathers taking sons to traditionally male-orientated sports (e.g., rugby or football) to bond over shared interests and give mothers a break from parenting.

The results from the current study suggest intervention studies should be developed to engage both parents, or specifically fathers, in taking the lead to support their children to be active, not necessarily focused on children and parents being active together, but rather on how parents can work together to schedule times for children to be active across the week in both structured and unstructured activities, and how parents can share the role between parenting partners. Table 4 summarises the key findings and implications for how parents can support child activity that have emerged from this study. These suggestions provide ways that researchers and policy makers can help parents to support their child's physical activity, through providing advice and encouragement to developing family physical activity plans.

For peer review only

495 **Table 4 Key findings and implications for how parents can support their child's physical activity**

Finding	Implication
Mothers primarily lead child physical activity during the week	Develop advice for mothers to help them facilitate their child's physical activity during busy weekdays (e.g., identifying times in the day for promoting activity, ideas for active games)
Engaging fathers to be involved in supporting child physical activity is important	Encourage fathers to see the important role they can play in supporting their child's activity
Children, possibly more so boys, are more active if both parents share the role of supporting child physical activity	Develop family physical activity plans (e.g., who can lead when) to encourage both parents to take an active role in supporting their child's physical activity
Parents can use physical activity time to bond over shared interests or engage in quality one-to-one time with children	Encourage parents to value physical activity time as a way to share interests and bond with children (e.g., promote physical activity as quality family time)
Some parents, possibly more so mothers, struggle for confidence when it comes to supporting child physical activity	Develop parental skills and confidence in supporting and facilitating child activity, and encourage parents to model the behaviours that they wish their child to adopt

For peer review only

For peer review only - http://bmjopen.bmj.com/site/about/guidelines.xhtml

Strengths and limitations

A main strength of the study is the mixed-methods approach, utilising both accelerometer-assessed physical activity from a large sample of 8-9-year-old children and semi-structured interview data with parents. This approach provides rich data about the gender roles associated with how parents support their child's activity. Another strength is that we interviewed a relatively large sample of parents, including 20 fathers, a group that are known to be difficult to engage in research.[46] Limitations of the study include its cross-sectional nature so causality could not be examined. In the main dataset, parents were primarily represented by mothers (72%), which is likely to have influenced how they responded to questions about who leads in supporting their child's activity. We had very limited power to explore gender interactions, thus whilst our results suggest that parent leadership to support their child's physical activity might have a strong positive impact on sons compared with daughters it would be wrong to conclude that from these data, and much larger independent studies are required to explore that further. Parental responses to our exposure questions provided no information on the type (quality or quantity) of their leadership role. Additionally, 279 participants were excluded from the study due to missing data, which may have resulted in sampling bias, because these participants differed from included participants in terms of their MVPA and household IMD score. This study is also drawn from the greater Bristol area (UK), and as such our ability to extend findings to other settings and countries is limited.

CONCLUSIONS

We found some evidence that parents share the role of supporting their children to be active. It is possible that mothers primarily lead child activity during the week, with the role shared

For peer review only - http://bmjopen.bmj.com/site/about/guidelines.xhtml

more equally on the weekend. Children are more active when parents share the responsibility of supporting their child's activity, but further large independent studies are required to replicate our findings and determine whether parental leadership has a stronger effect on sons than daughters. Future studies should also seek to engage more fathers, verify reports of who takes a leading role (for example through cross comparison of reports from each parent and the child or direct observation), and to collect information on the nature of leadership roles (quality and frequency).

ACKNOWLEDGEMENTS

This work was supported by grants from the British Heart Foundation (ref PG/11/51/28986 and SP 14/4/31123). DAL works in a unit that receives funding from the University of Bristol and UK Medical Research Council (MC_UU_1201/5); she is also a UK National Institute of Health and Research Senior Investigator (NF-SI-0166-10196). The funders had no involvement in data analysis, data interpretation or writing of the paper.

We would like to thank all of the families and schools that have taken part in the B-PROACT1V project. We would also like to thank all current and previous members of the research team who are not authors on this paper.

COMPETING INTERESTS

All authors have completed the ICMJE uniform disclosure form at www.icmje.org/coi_disclosure.pdf and declare: all authors had financial support from the British Heart Foundation for the submitted work; no financial relationships with any organisations that might have an interest in the submitted work in the previous three years; no other relationships or activities that could appear to have influenced the submitted work.

For peer review only - http://bmjopen.bmj.com/site/about/guidelines.xhtml

CONTRIBUTORS

Conception / design**:** RJ, ESM, JLT, DAL and SJS.

Quantitative and Qualitative data collection: ESM.

Data analysis / acquisition/ interpretation: ESM, RJ, ZT and DAL.

Drafting / revising critically for important content: All authors.

Final approval: All authors.

Accountability for study and manuscript: ESM, RJ.

DATA SHARING STATEMENT

The datasets generated during the current study are not publicly available as the project is ongoing and data are not ready for archiving. We will make quantitative data available to the wider research community once the project is complete in August 2019. Because of possible disclosure with qualitative data we will consider requests to use and further explore those data on a per request basis with an appropriate balance between sharing data as fully as possible whilst maintaining participant anonymity.

REFERENCES

1. Janssen I, LeBlanc AG. Systematic review of the health benefits of physical activity and fitness in school-aged children and youth. Int J Behav Nutr Phys Act 2010;**7**:40 doi: 10.1186/1479-5868-7-40.
2. Strong WB, Malina RM, Blimkie CJ, et al. Evidence based physical activity for school-age youth. J Pediatr 2005;**146**(6):732-7

For peer review only - http://bmjopen.bmj.com/site/about/guidelines.xhtml

3. Department of Health, Physical Activity, Health Improvement and Protection. Start Active, Stay Active: A report on physical activity from the four home countries' Chief Medical Officers. London, 2011.

4. Griffiths LJ, Cortina-Borja M, Sera F et al. How active are our children? Findings from the Millennium Cohort Study. BMJ Open 2013;**3**(8):e002893 doi: 10.1136/bmjopen-2013-002893.

5. Scholes S. Health Survey for England 2015: Physical activity in children. Health and Social Care Information Centre. London, 2016.

6. Cooper AR, Goodman A, Page AS, et al. Objectively measured physical activity and sedentary time in youth: the International children's accelerometry database (ICAD). Int J Behav Nutr Phys Act 2015;**12**:113 doi: 10.1186/s12966-015-0274-5.

7. Nader PR, Bradley RH, Houts RM, McRitchie SL, O'Brien M. Moderate-to-vigorous physical activity from ages 9 to 15 years. JAMA 2008;**300**(3):295-305.

8. Farooq MA, Parkinson KN, Adamson AJ, et al. Timing of the decline in physical activity in childhood and adolescence: Gateshead Millennium Cohort Study. Br J Sports Med 2017:1-6 doi:10.1136/bjsports-2016-096933.

9. Jago R, Solomon-Moore E, Macdonald-Wallis C, Sebire SJ, Thompson JL, Lawlor DA. Change in children's physical activity and sedentary time between Year 1 and Year 4 of primary school in the B-PROACT1V cohort. Int J Behav Nutr Phys Act 2017;**14**:33 doi: 10.1186/s12966-017-0492-0.

10. Patrick H, Hennessy E, McSpadden K, Oh A. Parenting styles and practices in children's obesogenic behaviors: scientific gaps and future research directions. Child Obes 2013;**9**(S1):S73-86 doi: 10.1089/chi.2013.0039.

For peer review only

For peer review only - http://bmjopen.bmj.com/site/about/guidelines.xhtml

11. Davison KK, Masse LC, Timperio A, et al. Physical activity parenting measurement and research: challenges, explanations, and solutions. Child Obes 2013;**9**(Suppl):S103-9 doi: 10.1089/chi.2013.0037.

12. Sleddens EF, Gerards SM, Thijs C, de Vries NK, Kremers SP. General parenting, childhood overweight and obesity-inducing behaviors: a review. Int J Pediatr Obes 2011;**6**(2-2):e12-27 doi: 10.3109/17477166.2011.566339.

13. O'Connor TM, Jago R, Baranowski T. Engaging parents to increase youth physical activity: a systematic review. Am J Prev Med 2009;**37**(2):141-9 doi: 10.1016/j.amepre.2009.04.020.

14. Jago R, Fox KR, Page AS, Brockman R, Thompson JL. Parent and child physical activity and sedentary time: do active parents foster active children? BMC Public Health 2010;**10**(1):194 doi: 10.1186/1471-2458-10-194.

15. Gustafson SL, Rhodes RE. Parental correlates of physical activity in children and early adolescents. Sports Med 2006;**36**(1):79-97.

16. Edwardson CL, Gorely T. Parental influences on different types and intensities of physical activity in youth: A systematic review. Psychol Sport Exerc 2010;**11**(6):522-35 doi: 10.1016/j.psychsport.2010.05.001.

17. Jago R, Sebire SJ, Wood L, et al. Associations between objectively assessed child and parental physical activity: a cross-sectional study of families with 5-6 year old children. BMC Public Health 2014;**14**:655 doi: 10.1186/1471-2458-14-655.

18. Jago R, Solomon-Moore E, Macdonald-Wallis C, Thompson JL, Lawlor DA, Sebire SJ. Association of parents' and children's physical activity and sedentary time in Year 4 (8-9) and change between Year 1 (5-6) and Year 4: a longitudinal study. Int J Behav Nutr Phys Act 2017;**14**:110 doi: 10.1186/s12966-017-0565-0.

For peer review only

19. Fuemmeler BF, Anderson CB, Mâsse LC. Parent-child relationship of directly measured physical activity. Int J Behav Nutr Phys Act 2011;8:17 doi: 10.1186/1479-5868-8-17.

20. Garriguet D, Colley R, Bushnik T. Parent-Child association in physical activity and sedentary behaviour. Health Reports 2017;**28**(6):3-11.

21. Erkelenz N, Kobel S, Kettner S, et al. Parental activity as influence on children's BMI percentiles and physical activity. J Sports Sci Med 2014;**13**:645-50.

22. Tate EB, Shah A, Jones M, et al. Toward a better understanding of the link between parent and child physical activity levels: The moderating role of parental encouragement. J Phys Act Health 2015;**12**:1238-44 doi: 10.1123/jpah.2014-0126.

23. Hennessy E, Hughes SO, Goldberg JP, et al. Parent-child interactions and objectively-measured child physical activity: a cross-sectional study. Int J Behav Nutr Phys Act 2010;**7**:71 doi: 10.1186/1479-5868-7-71.

24. Davison KK, Cutting TM, Birch LL. Parents' activity-related parenting practices predict girls' physical activity. Med Sci Sports Exerc 2003;**35**:1589-95 doi: 10.1249/01.MSS.0000084524.19408.0C.

25. Lloyd AB, Lubans DR, Plotnikoff RC, Collins CE, Morgan PJ. Maternal and paternal parenting practices and their influence on children's adiposity, screen-time, diet and physical activity. Appetite 2014;**79**:149-57 doi: 10.1016/j.appet.2014.04.010.

26. Davison KK, Gicevic S, Aftosmes-Tobio A, et al. Fathers' representation in observational studies on parenting and childhood obesity: a systematic review and content analysis. Am J Public Health 2016;**106**(11):e14-e21 doi: 10.2105/AJPH.2016.303391.

For peer review only - http://bmjopen.bmj.com/site/about/guidelines.xhtml

27. Zahra J, Sebire SJ, Jago R. “He’s probably more Mr. sport than me” – a qualitative exploration of mothers’ perceptions of fathers’ role in their children’s physical activity. BMC Pediatrics 2015;15:101 doi: 10.1186/s12887-015-0421-9.

28. Vollmer RL, Adamsons K, Gorin A, Foster JS, Mobley AR. Investigating the relationship of body mass index, diet quality, and physical activity level between fathers and their preschool-aged children. J Acad Nutr Diet 2015;115:919-26 doi: 10.1016/j.jand.2014.12.003.

29. Schoeppe S, Liersch S, Röbl M, Krauth C, Walter U. Mothers and fathers both matter: the positive influence of parental physical activity modelling on children’s leisure-time physical activity. Pediatr Exerc Sci 2016;**28**:466-72 doi: 10.1123/pes.2015-0236.

30. Morgan PJ, Collins CE, Plotnikoff RC, et al. The ‘healthy dads, healthy kids’ community randomized controlled trial: a community-based healthy lifestyle program for fathers and their children. Prev Med 2014;**61**:90-99 doi: 10.1016/j.ypmed.2013.12.019.

31. Morgan PJ, Lubans DR, Callister R, et al. The ‘healthy dads, healthy kids’ randomized controlled trial: efficacy of a healthy lifestyle program for overweight fathers and their children. Int J Obes 2011;**35**(3):436-47 doi: 10.1038/ijo.2010.151.

32. Eriksson M, Nordqvist T, Rasmussen F. Associations between parents’ and 12-year-old children’s sport and vigorous activity: the role of self-esteem and athletic competence. J Phys Act Health 2008;5(3):359-73 doi: 10.1123/jpah.5.3.359.

33. O’Loughlin J, Paradis G, Kishchuk N, Barnett T, Renaud L. Prevalence and correlates of physical activity behaviors among elementary schoolchildren in multi-ethnic, low income, inner-city neighborhoods in Montreal, Canada. Ann Epidemiol 1999;**9**(7):397-407.

For peer review only - http://bmjopen.bmj.com/site/about/guidelines.xhtml

34. Trost SG, Pate RR, Saunders R, Ward DS, Dowda M, Felton G. A prospective study of the determinants of physical activity in rural fifth-grade children. Prev Med 1997;**26**(2):257-63.

35. Trost SG, Pate RR, Ward DS, Saunders R, Riner W. Determinants of physical activity in active and low-active, sixth grade African-American youth. J Sch Health 1999;**69**(1):29-34.

36. Jago R, Thompson JL, Sebire SJ, et al. Cross-sectional associations between the screen-time of parents and young children: differences by parent and child gender and day of the week. Int J Behav Nutr Phys Act 2014;**11**:54 doi: 10.1186/1479-5868-11-54.

37. Evenson KR, Catellier DJ, Gill K, et al. Calibration of two objective measures of physical activity for children. J Sports Sci 2008;**26**:1557-65 doi: 10.1080/02640410802334196.

38. Creswell J, Plano Clark V. Designing and Conducting Mixed Methods Research. Thousand Oaks, CA: Sage, 2006.

39. Tashakkori A, Teddlie C. Handbook of Mixed Methods in Social & Behavioral Research. Thousand Oaks, CA: Sage, 2010.

40. Andrew S, Halcomb EJ. Mixed methods research is an effective method of enquiry for community health research. Contemp Nurse 2006;**23**(2):145-153.

41. Creswell, J. Research design: Qualitative, quantitative, and mixed methods approaches. Thousand Oaks, CA: Sage, 1997.

42. Elo S, Kyngäs H. The qualitative content analysis process. J Adv Nurs 2008;**62**(1): 107-15 doi: 10.1111/j.1365-2648.2007.04569.x.

For peer review only - http://bmjopen.bmj.com/site/about/guidelines.xhtml

43. Gale NK, Heath G, Cameron E, Rashid S, Redwood S. Using the framework method for the analysis of qualitative data in multi-disciplinary health research. BMC Med Res Methodol 2013;**13**:117 doi: 10.1186/1471-2288-13-117.

44. Anderson PM, Butcher KF. Childhood obesity trends and potential causes. Future Child 2006;**16**:19-45 doi: 10.1353/foc.2006.0001.

45. Laughlin L. Who's minding the kids? Child care arrangements: spring 2011. https://www.census.gov/prod/2013pubs/p70-135.pdf. Published April 2013.

46. Macfadyen A, Swallow V, Santacroce S, Lambert H. Involving fathers in research. J Spec Pediatr Nurs 2011;**16**(3):216-9 doi: 10.1111/j.1744-6155.2011.00287.x

Figure 1 Study flow of participants for the quantitative study

Figure 2 Study flow of participants for the qualitative study

For peer review only - http://bmjopen.bmj.com/site/about/guidelines.xhtml

Figure 1 Study flow of participants for the quantitative study

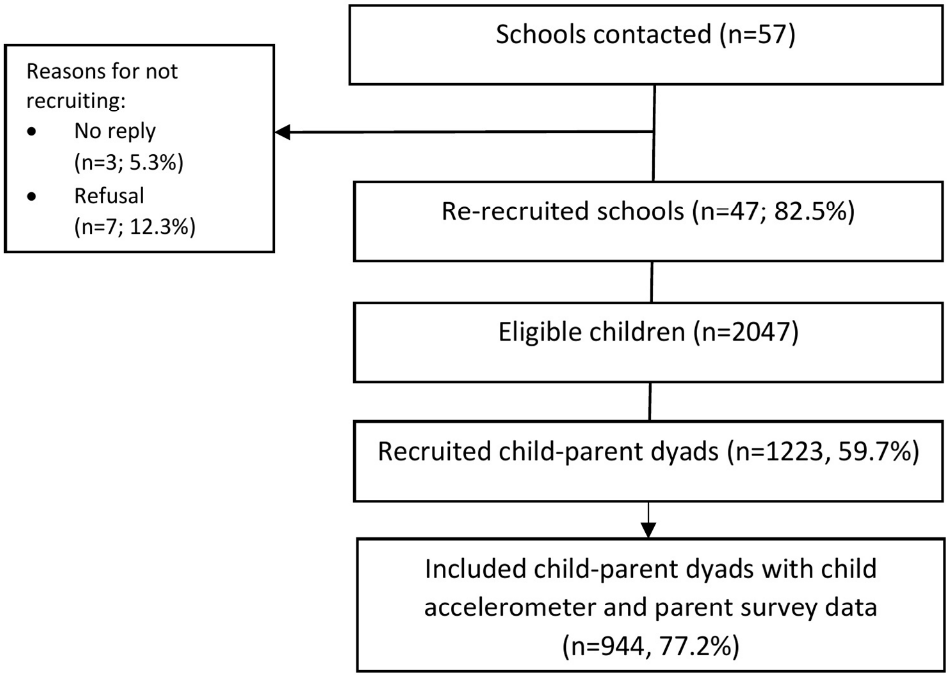

Figure 1 Study flow of participants for the quantitative study

132x104mm (300 x 300 DPI)

For peer review only - http://bmjopen.bmj.com/site/about/guidelines.xhtml

Figure 2 Study flow of participants for the qualitative study

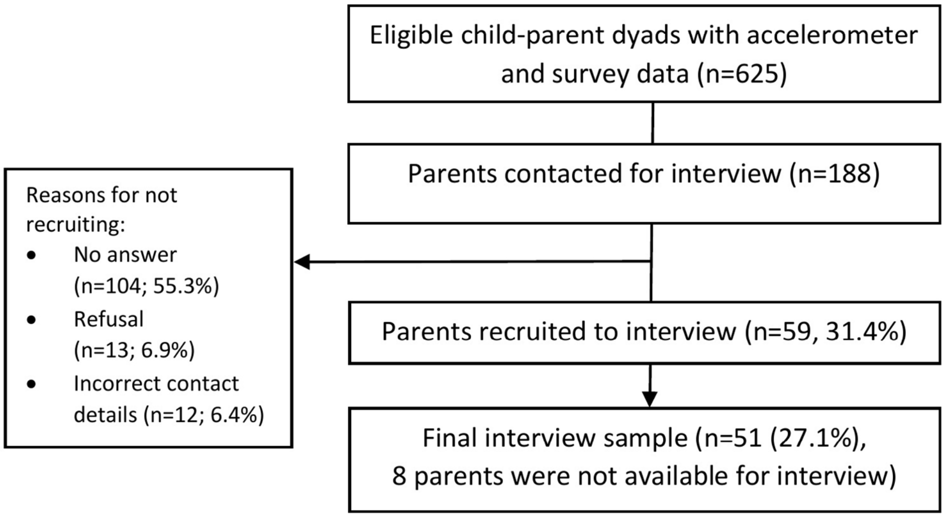

Figure 2 Study flow of participants for the qualitative study

132x81mm (300 x 300 DPI)

For peer review only

Table S1 Number of mothers and fathers who were eligible for and recruited to the interview study

		N parents eligible in each sub-group			N parents recruited in each sub-group		
**No.**	**Sub-group description**	**Total**	**Mothers**	**Fathers**	**Total**	**Mothers**	**Fathers**
1	Low MVPA, low SED boys	31	20	11	6	4	2
2	High MVPA, low SED boys	116	82	34	6	3	3
3	Low MVPA, high SED boys	67	51	16	7	5	2
4	High MVPA, high SED boys	63	48	15	6	3	3
5	Low MVPA, low SED girls	69	48	21	6	3	3
6	High MVPA, low SED girls	86	67	19	6	4	2
7	Low MVPA, high SED girls	138	111	27	7	4	3
8	High MVPA, high SED girls	55	37	18	7	5	2
	**Total**	**625**	**464**	**161**	**51**	**31**	**20**

For peer review only

Table S2 Frequency of reporting which parent leads child activity by parent and separately by child gender

		Parent gender			Child gender		
		Males (N=264) %	Females (N=680) %	Chi-squared p-value for difference	Boys (N=427) %	Girls (N=517) %	Chi-squared p-value for difference
**Takes lead role in child activity during the week**	Mother	26.9	57.4	<0.001	44.5	52.4	0.04
	Father	16.7	2.9		6.6	7.0	
	Both parents	56.4	39.7		48.9	40.6	
**Takes lead role in child activity at the weekend**	Mother	8.3	30.7	<0.001	21.1	27.3	0.02
	Father	26.9	14.1		20.8	15.1	
	Both parents	64.8	55.1		58.1	57.6	
**Who should take lead role in child activity**	Mother	1.1	6.9	0.001	4.2	6.0	0.07
	Father	1.5	0.7		1.6	0.4	
	Both parents	97.3	92.4		94.1	93.6	

For peer review only

Who leads and who supports? A cross-sectional mixed methods study of mothers' and fathers' support for child physical activity

STROBE Statement—checklist of items that should be included in reports of observational studies

	Item No	Recommendation
**Title and abstract**	1	(*a*) Indicate the study's design with a commonly used term in the title or the abstract Title Page
		(*b*) Provide in the abstract an informative and balanced summary of what was done and what was found – Pages 2-3
**Introduction**		
Background/rationale	2	Explain the scientific background and rationale for the investigation being reported – Pages 5-6
Objectives	3	State specific objectives, including any prespecified hypotheses – Page 6.
**Methods**		
Study design	4	Present key elements of study design early in the paper Pages 6-7.
Setting	5	Describe the setting, locations, and relevant dates, including periods of recruitment, exposure, follow-up, and data collection Pages 6-7.
Participants	6	(*a*) *Cohort study*—Give the eligibility criteria, and the sources and methods of selection of participants. Describe methods of follow-up<br>*Case-control study*—Give the eligibility criteria, and the sources and methods of case ascertainment and control selection. Give the rationale for the choice of cases and controls<br>*Cross-sectional study*—Give the eligibility criteria, and the sources and methods of selection of participants Page 7
		(*b*) *Cohort study*—For matched studies, give matching criteria and number of exposed and unexposed<br>*Case-control study*—For matched studies, give matching criteria and the number of controls per case
Variables	7	Clearly define all outcomes, exposures, predictors, potential confounders, and effect modifiers. Give diagnostic criteria, if applicable - Pages 7-9
Data sources/ measurement	8*	For each variable of interest, give sources of data and details of methods of assessment (measurement). Describe comparability of assessment methods if there is more than one group Pages 7-9
Bias	9	Describe any efforts to address potential sources of bias – Page 8
Study size	10	Explain how the study size was arrived at - Pages 7-8 (Figures 1 & 2)
Quantitative variables	11	Explain how quantitative variables were handled in the analyses. If applicable, describe which groupings were chosen and why - Pages 10-11
Statistical methods	12	(*a*) Describe all statistical methods, including those used to control for confounding – Pages 10-11
		(*b*) Describe any methods used to examine subgroups and interactions – Page 11
		(*c*) Explain how missing data were addressed - Pages 10-11
		(*d*) *Cohort study*—If applicable, explain how loss to follow-up was addressed<br>*Case-control study*—If applicable, explain how matching of cases and controls was

For peer review only

		addressed<br>*Cross-sectional study*—If applicable, describe analytical methods taking account of sampling strategy - Pages 10-11
		(*e*) Describe any sensitivity analyses – Page 11

Continued on next page

For peer review only

**Results**		
Participants	13*	(a) Report numbers of individuals at each stage of study—eg numbers potentially eligible, examined for eligibility, confirmed eligible, included in the study, completing follow-up, and analysed Table 1 (Figure 1 & 2)
		(b) Give reasons for non-participation at each stage - Figure 1 & 2
		(c) Consider use of a flow diagram – Figure 1 & 2
Descriptive data	14*	(a) Give characteristics of study participants (eg demographic, clinical, social) and information on exposures and potential confounders – Table 1
		(b) Indicate number of participants with missing data for each variable of interest - Table 1
		(c) *Cohort study*—Summarise follow-up time (eg, average and total amount)
Outcome data	15*	*Cohort study*—Report numbers of outcome events or summary measures over time
		Case-control study—Report numbers in each exposure category, or summary measures of exposure
		Cross-sectional study—Report numbers of outcome events or summary measures Table 1
Main results	16	(*a*) Give unadjusted estimates and, if applicable, confounder-adjusted estimates and their precision (eg, 95% confidence interval). Make clear which confounders were adjusted for and why they were included – Tables 2-3
		(*b*) Report category boundaries when continuous variables were categorized - Tables 2-3
		(*c*) If relevant, consider translating estimates of relative risk into absolute risk for a meaningful time period Discussion – N/A
Other analyses	17	Report other analyses done—eg analyses of subgroups and interactions, and sensitivity analyses – Supplementary tables
**Discussion**		
Key results	18	Summarise key results with reference to study objectives – Page 22
Limitations	19	Discuss limitations of the study, taking into account sources of potential bias or imprecision. Discuss both direction and magnitude of any potential bias - Page 25
Interpretation	20	Give a cautious overall interpretation of results considering objectives, limitations, multiplicity of analyses, results from similar studies, and other relevant evidence – Page 25-26
Generalisability	21	Discuss the generalisability (external validity) of the study results Page 25
**Other information**		
Funding	22	Give the source of funding and the role of the funders for the present study and, if applicable, for the original study on which the present article is based – Page 26

*Give information separately for cases and controls in case-control studies and, if applicable, for exposed and unexposed groups in cohort and cross-sectional studies.

Note: An Explanation and Elaboration article discusses each checklist item and gives methodological background and published examples of transparent reporting. The STROBE checklist is best used in conjunction with this article (freely available on the Web sites of PLoS Medicine at http://www.plosmedicine.org/, Annals of Internal Medicine at http://www.annals.org/, and Epidemiology at http://www.epidem.com/). Information on the STROBE Initiative is available at www.strobe-statement.org.

For peer review only

COREQ (COnsolidated criteria for REporting Qualitative research) Checklist

A checklist of items that should be included in reports of qualitative research. You must report the page number in your manuscript where you consider each of the items listed in this checklist. If you have not included this information, either revise your manuscript accordingly before submitting or note N/A.

Topic	Item No.	Guide Questions/Description	Reported on Page No.
**Domain 1: Research team and reflexivity**			
Personal characteristics			
Interviewer/facilitator	1	Which author/s conducted the interview or focus group?	Page 27
Credentials	2	What were the researcher's credentials? E.g. PhD, MD	Page 9
Occupation	3	What was their occupation at the time of the study?	Page 9
Gender	4	Was the researcher male or female?	Page 9
Experience and training	5	What experience or training did the researcher have?	Page 9
Relationship with participants			
Relationship established	6	Was a relationship established prior to study commencement?	Pages 8-9
Participant knowledge of the interviewer	7	What did the participants know about the researcher? e.g. personal goals, reasons for doing the research	N/A
Interviewer characteristics	8	What characteristics were reported about the inter viewer/facilitator? e.g. Bias, assumptions, reasons and interests in the research topic	N/A
**Domain 2: Study design**			
Theoretical framework			
Methodological orientation and Theory	9	What methodological orientation was stated to underpin the study? e.g. grounded theory, discourse analysis, ethnography, phenomenology, content analysis	Page 11
Participant selection			
Sampling	10	How were participants selected? e.g. purposive, convenience, consecutive, snowball	Pages 8-9
Method of approach	11	How were participants approached? e.g. face-to-face, telephone, mail, email	Page 9
Sample size	12	How many participants were in the study?	Page 9
Non-participation	13	How many people refused to participate or dropped out? Reasons?	Page 9 (Fig 2)
Setting			
Setting of data collection	14	Where was the data collected? e.g. home, clinic, workplace	Page 8
Presence of non-participants	15	Was anyone else present besides the participants and researchers?	N/A
Description of sample	16	What are the important characteristics of the sample? e.g. demographic data, date	Pages 8-9, 12 (
Data collection			
Interview guide	17	Were questions, prompts, guides provided by the authors? Was it pilot tested?	Page 9
Repeat interviews	18	Were repeat inter views carried out? If yes, how many?	N/A
Audio/visual recording	19	Did the research use audio or visual recording to collect the data?	Page 9
Field notes	20	Were field notes made during and/or after the inter view or focus group?	N/A
Duration	21	What was the duration of the inter views or focus group?	Page 12
Data saturation	22	Was data saturation discussed?	Page 9
Transcripts returned	23	Were transcripts returned to participants for comment and/or	N/A

For peer review only

For peer review only - http://bmjopen.bmj.com/site/about/guidelines.xhtml

Topic	Item No.	Guide Questions/Description	Reported on Page No.
		correction?	
**Domain 3: analysis and findings**			
Data analysis			
Number of data coders	24	How many data coders coded the data?	Page 11
Description of the coding tree	25	Did authors provide a description of the coding tree?	N/A
Derivation of themes	26	Were themes identified in advance or derived from the data?	Page 11
Software	27	What software, if applicable, was used to manage the data?	Page 11
Participant checking	28	Did participants provide feedback on the findings?	N/A
Reporting			
Quotations presented	29	Were participant quotations presented to illustrate the themes/findings? Was each quotation identified? e.g. participant number	Pages 14-17
Data and findings consistent	30	Was there consistency between the data presented and the findings?	Pages 14-17
Clarity of major themes	31	Were major themes clearly presented in the findings?	Pages 14-17
Clarity of minor themes	32	Is there a description of diverse cases or discussion of minor themes?	Pages 14-17

Developed from: Tong A, Sainsbury P, Craig J. Consolidated criteria for reporting qualitative research (COREQ): a 32-item checklist for interviews and focus groups. *International Journal for Quality in Health Care*. 2007. Volume 19, Number 6: pp. 349 – 357

Once you have completed this checklist, please save a copy and upload it as part of your submission. DO NOT include this checklist as part of the main manuscript document. It must be uploaded as a separate file.

For peer review only

The roles of mothers and fathers in supporting child physical activity: a cross-sectional mixed-methods study

Journal:	*BMJ Open*
Manuscript ID	bmjopen-2017-019732.R1
Article Type:	Research
Date Submitted by the Author:	27-Oct-2017
Complete List of Authors:	Solomon-Moore, Emma; Univ Bristol<br>Toumpakari, Zoi; University of Bristol, Centre for Exercise, Nutrition and Health Sciences<br>Sebire, Simon; University of Bristol<br>Thompson, Janice; The University of Birmingham, School of Sport & Exercise Sciences<br>Lawlor, Debbie; Population Health Sciences, Bristol Medical School, MRC Integrative Epidemiology Unit at the University of Bristol<br>Jago, Russ; University of Bristol, Centre for Exercise, Nutrition and Health Sciences
**Primary Subject Heading**:	Public health
Secondary Subject Heading:	Paediatrics
Keywords:	Physical activity, children, parents, gender, mixed-methods

SCHOLARONE™ Manuscripts

The roles of mothers and fathers in supporting child physical activity: a cross-sectional mixed-methods study

Emma Solomon-Moore [1], Zoi Toumpakari [1], Simon J. Sebire [1], Janice L. Thompson [2], Deborah A. Lawlor [3, 4], and Russell Jago [1$]

[1] Centre for Exercise, Nutrition & Health Sciences, School for Policy Studies, University of Bristol, 8 Priory Road, Bristol, BS8 1TZ, UK.

[2] School of Sport, Exercise and Rehabilitation Sciences, University of Birmingham, Birmingham, B15 2TT, UK.

[3] MRC Integrative Epidemiology Unit at the University of Bristol, Oakfield House, Oakfield Grove, Bristol, BS8 2BN, UK.

[4] Population Health Sciences, Bristol Medical School, University of Bristol, Canynge Hall, Whiteladies Road, Bristol, BS8 2PS, UK.

[$] **Corresponding author:** Professor Russell Jago, Centre for Exercise, Nutrition & Health Sciences, School for Policy Studies, University of Bristol, 8 Priory Road, Bristol, BS8 1TZ, UK. Email: russ.jago@bristol.ac.uk; Telephone: +44 (0)117 9546603

Email addresses

ESM: Emma.solomon@bristol.ac.uk

ZT: Z.Toumpakari@bristol.ac.uk

SJS: simon.sebire@bristol.ac.uk

JLT: j.thompson.1@bham.ac.uk

DAL: d.a.lawlor@bristol.ac.uk

RJ: russ.jago@bristol.ac.uk

Word count: **Manuscript** (excluding tables) = 4734 words **Abstract** = 300 words

For peer review only - http://bmjopen.bmj.com/site/about/guidelines.xhtml

For peer review only

ABSTRACT

Objectives: Examine the extent parent gender is associated with supporting children's physical activity.

Design: Cross-sectional mixed-methods study.

Setting: 47 primary schools located in Bristol (UK).

Participants: 944 8-9-year-old children and one of their parents provided quantitative data; 51 parents (20 fathers) were interviewed.

Methods: Children wore an accelerometer and mean minutes of moderate-to-vigorous-intensity physical activity (MVPA) per day, counts per minute (CPM), and achievement of national MVPA guidelines were derived. Parents reported who leads in supporting child activity during the week and weekend. Linear and logistic regression examined the association between gender of parent who supports child activity and child physical activity. For the semi-structured telephone interviews, inductive and deductive content analysis were used to explore the role of gender in how parents support child activity.

Results: Parents appeared to have a stronger role in supporting boys to be more active, than girls, and the strongest associations were when they reported that both parents had equal roles in supporting their child. For example, compared with the reference of female/mother support, equal contribution from both parents during the week was associated with boys doing 5.9 (95% CI: 1.2 to 10.6) more minutes of MVPA per day, and more CPM when both parents support on weekday and weekends (55.1 [14.3 to 95.9] and 52.8 [1.8 to 103.7], respectively). Associations in girls were weaker and sometimes in the opposite direction but there was no strong statistical evidence for gender interactions. Themes emerged from the qualitative data, specifically; parents proactively supporting physical activity equally, mothers supporting during the week, families getting together at weekends, families doing

For peer review only - http://bmjopen.bmj.com/site/about/guidelines.xhtml

activities separately due to preferences, and parents using activities to bond one-to-one with children.

Conclusions: Mothers primarily support child activity during the week. Children, possibly more so boys, are more active if both parents share the supporting role.

Key words: Physical activity, children, parents, gender, mixed-methods

For peer review only

For peer review only - http://bmjopen.bmj.com/site/about/guidelines.xhtml

ARTICLE SUMMARY

Strengths and limitations of this study

Strengths

- Mixed-methods study.
- Accelerometer data from a large sample of 8-9-year-old children.
- Semi-structured telephone interviews with 51 parents, including 20 fathers.

Limitations

- Cross-sectional study design from a single UK region.
- The measurement of parental support of child physical activity would be strengthened by collecting data from both parents and information on the quality and quantity of support.

For peer review only

For peer review only - http://bmjopen.bmj.com/site/about/guidelines.xhtml

INTRODUCTION

Children who are physically active are at a lower risk of obesity, high blood pressure, metabolic syndrome, and depression.[1 2] The UK Government recommends that children and young people aged 5 to 18 years should engage in at least 60 minutes of moderate-to-vigorous-intensity physical activity (MVPA) every day.[3] However, data from the nationally representative Millennium cohort showed that only 51% of 7-8 year olds met the recommendation.[4] Physical activity declines throughout childhood and adolescence, with boys being more active than girls at all ages.[4-9] Thus, in order to develop effective means of increasing child physical activity, there is a need to understand the factors that influence behaviour.

Parents act as gatekeepers to children's activity,[10] and can play an important role in increasing their child's physical activity.[11-13] For instance, parents can influence their child's activity by being active with their child, role-modelling active behaviour, and/or by facilitating physical activity for their child (logistic support).[13-16] Studies examining associations between parent and child physical activity behaviour have yielded mixed results.[14 17-20] A growing body of research has shown that providing logistic support is associated with increased physical activity,[21-23] and therefore, may be the most important source of parental influence on children's activity.

The gender of the parent who takes the lead in supporting child activity could be an important influence on children's activity levels. Traditional gender roles comprised of the public sphere (employment, education, politics) being dominated by men and the private sphere (home, family) being exclusively the realm of women.[24] However, these traditional roles

For peer review only - http://bmjopen.bmj.com/site/about/guidelines.xhtml

have been shifting, as explained by the gender revolution framework,[25] whereby men's attitudes have become much more accepting of gender equality in the family,[26] particularly in caring for children.[27] It is not clear what the current role gender plays in parental physical activity support. Several studies suggest that mothers play a larger role in the logistical planning of children's physical activity, while fathers are more likely to model physical activity.[28 29] However, most studies in this area have focused on the mother-child relationship, and relatively little attention has been paid to the role of fathers.[30] From qualitative interviews with parents of 5-6-year-old children in the B-Proact1v study, we found evidence that fathers play a key role in promoting children's physical activity, influencing their choices and behaviours,[31] a finding replicated in other studies.[32 33] The Healthy Dads, Healthy Kids intervention demonstrated that engaging fathers in physical activity with their children can promote increased physical activity among children.[34 35] Data from the B-Proact1v interviews suggest that fathers may take more responsibility for their son's physical activity (e.g., taking their son to sports clubs), and mothers with their daughter's activity.[31] To date, there is inconsistent evidence regarding whether gender-specific parental influence (i.e., mothers with daughters and fathers with sons) is stronger than cross-gender parental influence (i.e., mothers with sons and fathers with daughters) on children's physical activity.[28 36-39] Therefore, a greater understanding is needed about the role gender plays in how parents support their child to be active, and if this varies by child gender.

The aim of this mixed-methods study was to examine parent gender, in terms of which parent supports their child to be active, and its association with child physical activity. A secondary aim was to discover if these associations varied by child gender.

For peer review only - http://bmjopen.bmj.com/site/about/guidelines.xhtml

METHODS

Data are from the longitudinal B-Proact1v study, which aimed to examine factors associated with children's and parents' physical activity, sedentary time and screen-viewing behaviours. The study has been described in detail elsewhere.[9 17 40] Briefly, in 2012 and 2013, data were collected from 1299 Year 1 children (5-6 years old) from 57 primary schools across Bristol, UK. Between March 2015 and July 2016, 47 of the original schools were re-recruited and data were collected from 1223 Year 4 children (8-9 years old). One of the children's parents were also recruited to the study. The current study used a mixed-methods design, incorporating cross-sectional data from the Year 4 assessments, for the 944 children and parents who provided valid child accelerometer data and complete parent questionnaire data for questions on child and parent demographics and gender roles associated with supporting child activity (Figure 1), with qualitative data via semi-structured telephone interviews from a sub-sample of 51 parents (details below; Figure 2). The current study incorporated a convergent parallel mixed-methods design. Quantitative data were collected prior to qualitative data collection, but the analyses and interpretation were conducted in parallel.[41] The study received ethical approval from the School for Policy Studies Ethics Committee at the University of Bristol, and written parent consent was received for all participants.[42]

Accelerometer data

Children wore a waist-worn ActiGraph wGT3X-BT accelerometer for five days including two weekend days. Waist-worn accelerometers have been demonstrated to be valid for measuring physical activity in children.[43 44] Accelerometer data were processed using Kinesoft (v3.3.75; Kinesoft, Saskatchewan, Canada), and were included in the primary

For peer review only - http://bmjopen.bmj.com/site/about/guidelines.xhtml

analyses if children provided at least three days of valid data (including at least one weekend day). A valid day was defined as at least 500 minutes of data after excluding intervals of ≥60 minutes of zero counts, allowing up to two minutes of interruptions. Minutes spent in MVPA were derived using population-specific cut points for children.[45] In a comparative study with other widely-used accelerometer cut points, the Evenson thresholds,[45] (in which stair climbing and brisk walking corresponded to moderate-intensity physical activity) were shown to provide the most accurate assessments of children's energy expenditure.[46] Mean accelerometer counts per minute (CPM), and a binary variable indicating whether the child's average daily MVPA was greater than the 60 minutes per day recommended by the UK government,[3] were also derived.

Parent support variables

To understand the gender roles associated with parents supporting their child's activity, parents were asked three questions via a questionnaire: a) "In your family who takes the lead role in supporting your Year 4 child to be active during the week?", b) "In your family who takes the lead role in supporting your Year 4 child to be active at the weekend?" and c) "Who do you think should take the lead role in supporting your Year 4 child to be active?". Each question had three response options: "Mother/Female care-giver", "Father/Male care-giver" or "About the same" for questions a) and b), and "Should be shared" for question c).

Demographic information

Parents provided demographic information via a questionnaire, including parent and child gender, date of birth, and ethnic origin. Where children's date of birth was missing (21% of children) they were assigned the median age of 9.0 years (as the children were all in the same

For peer review only

For peer review only - http://bmjopen.bmj.com/site/about/guidelines.xhtml

school year with a maximum age difference between the youngest and oldest of just under 12-months legally possible). As an indicator of socio-economic status, Indices of Multiple Deprivation (IMD) scores, based upon the English Indices of Deprivation,[47] were assigned to each child based on their reported home postcode, where higher scores indicate greater levels of deprivation. IMD scores provide a set of relative measures of deprivation for lower-layer super output areas across England, based on seven different domains of deprivation: income deprivation; employment deprivation; education, skills and training deprivation; health deprivation and disability; crime; barriers to housing and services; and living environment deprivation. Child height, weight and blood pressure were also measured.

Interview data

During consent procedures, parents were informed that they may be re-contacted to take part in a telephone interview. Only families with complete data for all measures (accelerometer and questionnaire data, child height, weight and blood pressure) were included in the interview sample (N=625, of which 161 (25.8%) had data from fathers). This sample was stratified according to the child's MVPA minutes per day (dichotomised around the study median: 57.5 minutes), sedentary minutes per day (dichotomised around the median: 434.6 minutes), and by child gender. This produced eight sub-groups (1 = low MVPA, low sedentary time boys; and 8 = high MVPA, high sedentary time girls; Table S1). The order in which parents were invited to participate in an interview was randomised within each sub-group. Contact attempts were made with 188 parents in total, of which 59 (31.4%) initially agreed to participate in an interview, and 51 (27.1%) completed an interview (Figure 2). Interviews were audio-recorded and continued until theoretical saturation was reached for the entire sample and the sub-groups. Parents were invited to participate by telephone between

For peer review only - http://bmjopen.bmj.com/site/about/guidelines.xhtml

July and October 2016, and interviews were conducted at the interviewee's convenience (37 during weekday daytimes (72.5%), 13 during weekday evenings (25.5%), and 1 on a weekend evening (2%)). Participants were sent a £10 high street shopping voucher as a thank you for their time.

An interview guide was developed and refined by the research team based on identifying gaps in current knowledge and guided by the Year 1 B-Proact1v quantitative and qualitative findings. This included questions relating to a variety of topics, including parents' perceptions of their child's physical activity and screen-viewing behaviours,[48] strategies for managing these behaviours,[49 50] understanding what has changed regarding these behaviours,[17 40] and understanding how family dynamics influence children's physical activity.[51] The need to engage more fathers in research was also identified as a priority.[31 51] Questions were posed in a non-leading manner to allow participants to shape the direction of the interview, and issues that emerged were probed. Interviews were conducted by two female researchers (qualified to at least MSc level) who were trained in conducting qualitative interviews.

Data analysis

Quantitative data

Means, proportions and Chi Square statistics were used to examine the distributions of exposures, outcomes and co-variates between participants included and excluded in this study, and between child and parent gender. Nearly all parents reported that both parents "*should take the lead*" in supporting their child's activity (93.8%), therefore we could not explore the association of parental attitudes towards who should support child physical

For peer review only - http://bmjopen.bmj.com/site/about/guidelines.xhtml

activity, as numbers were too small in the mother or father only categories. We used linear regression models to examine the associations of parent support of child activity during the week and weekend with the child's MVPA minutes per day and CPM, and logistic regression models to examine associations with achievement of the MVPA guideline. Models were adjusted for child age, gender of parent providing the information on support, and household IMD score. Robust standard errors were used to account for the clustering of children in schools for all models. Models were examined for all children, and separately for boys and girls. Combined Wald tests were used to test for evidence of interaction between child gender and the exposure of interest. All analyses were performed in Stata version 14.0 (StataCorp, 2015).

Qualitative data

Interviews were transcribed verbatim and anonymised before being entered into QSR NVivo 10 (QSR International, Warrington UK) to facilitate analysis. Using the framework method, thematic content analysis was performed by two researchers, enabling themes to develop both inductively from the accounts (experiences and views) of participants and deductively from existing literature.[52 53] Analysis involved several phases: familiarisation, coding, developing a framework, applying the framework, charting data into the framework matrix, and interpretation. During familiarisation, transcripts were thoroughly read and re-read independently by two researchers to immerse themselves in the data. After discussion between the two researchers, an initial coding frame was developed and applied to the data based on pre-existing ideas, and was refined throughout the process to allow for the inductive emergence of additional themes. The two researchers met regularly to ensure accuracy and consistency. Any disagreements that occurred during coding were discussed with additional

For peer review only

members of the research team to ensure consensus, and no disagreements remained unsolved. Hierarchies of categories were created and summarised, and brief summaries, mind maps, and representative quotes for each category were abstracted for reporting purposes. The final quotes were selected as they are illustrative of several responses given by parents.

RESULTS

Participant characteristics

The characteristics of the participants included and excluded from the quantitative dataset, and from the subset of interview participants, are shown in Table 1. Of the 944 included families, the majority (680 (72%)) had data from a mother/female care giver, with 264 (28%) from fathers/male care givers. Children excluded due to missing data were more likely to be deprived and did less minutes of MVPA per day, but were otherwise similar to the included dataset. Of the interview participants (N=51), 31 were mothers and 20 were fathers, with an average age of 41.2 (SD: 4.5) years, and 94.1% were White British. The interview participants were generally comparable to the main dataset, but tended to be less deprived. Interview participants were also more likely to be fathers and have less active children compared to the main dataset. The average interview duration was 34.4 minutes (SD: 8.0 minutes, range: 18 to 55 minutes).

For peer review only - http://bmjopen.bmj.com/site/about/guidelines.xhtml

Characteristic	Included (N=944)	Excluded		*p*	Interview sample (N=51)
	Mean (SD) or %	N	Mean (SD) or %		Mean (SD) or %
Child MVPA (mins/day)	62.8 (22.8)	209	58.6 (21.4)	0.01	58.3 (17.4)
Accelerometer counts per minute	620.4 (203.2)	209	609.0 (208.8)	0.46	573.2 (142.0)
Met MVPA guidelines (≥60 mins/day)		209		0.06	
No	52.0		59.3		58.8
Yes	48.0		40.7		41.2
Child gender		279		0.73	
Boy	45.2		46.4		49.0
Girl	54.8		53.6		51.0
Age of child (years)	9.03 (0.46)	279	9.04 (0.49)	0.91	8.95 (0.37)
Household IMD[b] score	15.1 (13.6)	248	18.8 (15.5)	<0.001	11.5 (9.7)
Supports child activity during the week		39		0.92	
Mother	48.8		48.7		43.1
Father	6.8		5.1		9.8
Both parents	44.4		46.2		47.1
Supports child activity at the weekend		37		0.35	
Mother	24.5		32.4		23.5
Father	17.7		21.6		23.5
Both parents	57.8		45.9		52.9
Who should support child PA		38		0.64	
Mother	5.2		2.6		3.9
Father	1.0		0.0		3.9
Both parents	93.8		97.4		92.2
Parent gender		41		0.24	
Male	28.0		19.5		39.2
Female	72.0		80.5		60.8
Parent ethnic origin		53		0.52	
White British	89.2		91.3		94.1

For peer review only - http://bmjopen.bmj.com/site/about/guidelines.xhtml

279 **Table 1 Descriptive characteristics of the main study sample (N=944) and subset of interview participants (N=51)**

280 MVPA: Moderate-to-vigorous physical activity; IMD: Index of multiple deprivation; a higher value indicates greater deprivation

For peer review only

For peer review only - http://bmjopen.bmj.com/site/about/guidelines.xhtml

Supplementary Table 2 shows the gender of the parent who reportedly supports child physical activity by parent and child gender. Mothers reported that typically they led in supporting their child's physical activity during the week, whereas fathers generally reported that duties were shared between parents. Most mothers and fathers reported that both parents shared the role of supporting their child's activity at the weekend, however, 31% of mothers and 27% of fathers, respectively, reported that they led child activity.

The interview data generally supported this, with several mothers stating that they support their child to be active during the week out of necessity because fathers were working long hours or late into the evening. Some mothers also reported that they try to get the whole family together to do activities at the weekend, although this isn't always the norm.

"On a weekday it's just, you know, every night we've got one or the other [children] have got a club on so it's just finish school and then me taking the children to their various clubs and then coming home and it's, erm, you know, pretty much get ready for bedtime ... Weekends, yeah, we try to do stuff as a family." [Int 14, Mother, Girl, 63 MVPA minutes/day, Mother supports weekday PA, Both parents support weekend PA]

"We like to do things as a family when we can; it's just all being around. My husband works quite late hours and things like that ... He's, he's home when they're going to bed usually ... but like last Sunday, we all went swimming together as a family thing... but that isn't – to be honest, that isn't like, isn't like we would do that every weekend or anything" [Int 35, Mother, Girl, 72 MVPA minutes/day, Mother supports weekday PA, Both parents support weekend PA]

For peer review only - http://bmjopen.bmj.com/site/about/guidelines.xhtml

Some parents indicated that they share the responsibility of supporting child physical activity, due to sharing an appreciation for the benefits of physical activity or because they value physical activity and feel a moral responsibility to fit activity in to the realities of life.

"I'm active, my husband's active. And so, you know, we cascade that if you like down to the children so we, we don't really sit around at all, we're very active and on the go…" [Int 3, Mother, Son, 59 MVPA minutes/day, Both parents support weekday and weekend PA]

"Actively we are trying to get the children involved in the various, activities like where there's after-school or a swimming lesson or they are going to join Scouts, which will be helpful for them in the long run… So, so we, we are encouraging them to get involved in outdoor activities as much as possible." [Int 1, Father, Son, 76 MVPA minutes/day, Both parents support weekday and weekend PA]

"So wherever we can we'll always try and do the right thing [physical activity] and, you know, sometimes if it's not taking the car and it's walking distance we'll try and walk, and things like that.." [Int 18, Father, Son, 86 MVPA minutes/day, Father supports weekday and weekend PA]

A few parents reported sharing the responsibility of supporting child physical activity, but also doing activities separately due to child preferences. Examples included fathers and sons using physical activity time to bond over shared interests, while also giving mothers a respite for some "me time", or parents taking children to separate activities to appease child

For peer review only - http://bmjopen.bmj.com/site/about/guidelines.xhtml

preferences, avoid conflict, and/or facilitate parent-child one-on-one time irrespective of gender.

"We like going about walking as a family. Well, I say me and my husband do and we drag the kids along, but, you know, it's just getting some fresh air, but the boys have their own interests as well, such as the rugby or football which my husband takes the boys to. I have a bit of 'me time' when they go off to do that so, you know, it's a mix, I think." [Int 32, Mother, Girl, 86 MVPA minutes/day, Both parents support weekday and weekend PA]

"I would like to do a little bit more with them but because my son doesn't like what [child] likes and I would like to take them swimming together a little bit more so we can all go and do swimming but because he doesn't like it; we kind of end up two of us doing it and two of us not doing it" [Int 29, Mother, Girl, 56 MVPA minutes/day, Both parents support weekday and weekend PA]

"I've said I might take him mountain biking this Sunday because I see that as exercise for him but also one to one. So, he's getting that, the benefit of obviously exercise, the sport that he actually really loves and is getting one to one time with a parent where, you know, it's hard isn't it, when there's other siblings" [Int 3, Mother, Son, 59 MVPA minutes/day, Both parents support weekday and weekend PA]

In the quantitative dataset, parents of girls tended to report that mothers take the lead in supporting their daughter's activity during the week, while parents of boys tended to report that the role was shared between both parents. Parents of boys and girls generally reported

For peer review only

For peer review only - http://bmjopen.bmj.com/site/about/guidelines.xhtml

that they shared the responsibility of supporting child activity at the weekend, although parents of girls were more likely to report that mothers supported their daughter's weekend activity.

In contrast, the interview data revealed a mix of gender patterns associated with supporting child physical activity, not just mothers supporting daughters and fathers supporting sons. Some fathers reported that they supported their daughter's physical activity through chauffeuring them to sports clubs, and expressed that they do so not just for logistical reasons, but also because they get real enjoyment from watching. A few mothers reported a lack of confidence in their own physical activity, because they aren't "naturally sporty" and so they tend to let fathers take the lead in supporting child physical activity.

"Yeah, she's been playing football for two and a half seasons now … and she's passionate about that. So I'm just a sort of chauffeur dad … that stands on the touchline in the cold windy rain. I enjoy that." [Int 51, Father, Girl, 71 MVPA minutes/day, Father supports weekday and weekend PA]

"Not that confident cause, like I say, I'm not actually naturally sporty or active. So it would be something that we would probably do as a family with their dad, and we could do it together……He's more confident, yeah, and he's more knowledgeable really with all that kind of stuff. And he's a – and he's the kind of person that's very much into, 'Come on, let's give it a go. Let's try and see. We might really enjoy it,' whereas I'm a bit more like, 'Oh no, don't make me do this. I'm really nervous.' And so I would probably shy away from it." [Int

For peer review only - http://bmjopen.bmj.com/site/about/guidelines.xhtml

24, Mother, Girl, 43 MVPA minutes/day, Mother supports weekday PA, Father supports weekend PA]

Associations of who supports child activity with child physical activity variables

Table 2 shows the mean difference in child MVPA minutes per day by which parent/s take the lead in supporting child activity during the week and weekend. Compared to reporting that mothers support child activity (reference group), reporting that parents share the role of supporting child activity during the week was associated with children doing, on average, an additional 3.5 minutes of MVPA per day. When examined separately by child gender, parents sharing the role of supporting child activity during the week was associated with, on average, an additional 5.9 minutes of MVPA per day for boys, and 0.4 minutes per day for girls, with no strong statistical evidence of a difference between boys and girls ($P_{interaction}$ = 0.34). Fathers taking the lead in supporting child activity (compared to mothers) was more weakly associated with child MVPA, with an inverse (rather than positive) association for girls, but again with no strong statistical evidence for gender interaction. Associations for parent support of child physical activity during the weekend showed very similar patterns to those for weekday activity, but were somewhat weaker in magnitude. In general, the patterns of association with achieving MVPA recommendations were similar to what was found for MVPA as a continuous measure, including point estimates suggesting weaker or inverse effects in girls but no evidence of gender interaction (Table 3). The one exception was that fathers supporting activity at weekends had a similar magnitude of effect as both parents being supporters.

For peer review only - http://bmjopen.bmj.com/site/about/guidelines.xhtml

399 The mean difference in children's CPM by parent/s who supports child activity during the week also showed a similar pattern to that seen for time spent in MVPA (Table 2).

For peer review only - http://bmjopen.bmj.com/site/about/guidelines.xhtml

Table 2 Mean difference in the children's average MVPA minutes per day and accelerometer counts per minute associated with gender of parent who supports physical activity during the week and weekend (N=944)

Exposure		Moderate-to-vigorous physical activity (minutes/day): mean difference (95% confidence interval)			P for gender interaction
		All (N=944)	Boys (N=427)	Girls (N=517)	
Supports child activity during week	Mother (ref)	0	0	0	0.34
	Father	0.3 (-5.7, 6.3)	8.1 (-1.7, 17.9)	-3.7 (-10.4, 2.9)	
	Both parents	3.5 (0.6, 6.5)	5.9 (1.2, 10.6)	0.4 (-3.0, 3.8)	
Supports child activity at the weekend	Mother (ref)	0	0	0	0.22
	Father	1.7 (-2.8, 6.2)	5.7 (-1.5, 12.9)	-3.4 (-8.5, 1.7)	
	Both parents	2.4 (-1.1, 5.9)	4.5 (-1.4, 10.3)	0.7 (-3.0, 4.4)	
**Exposure**		**Accelerometer counts per minute: mean difference (95% confidence interval)**			
		**All (N=944)**	**Boys (N=427)**	**Girls (N=517)**	**P for gender interaction**
Supports child activity during week	Mother (ref)	0	0	0	0.61
	Father	0.7 (-51.7, 53.2)	56.7 (-28.8, 142.1)	-22.8 (-86.7, 41.1)	
	Both parents	28.0 (2.0, 54.0)	55.1 (14.3, 95.9)	2.8 (-29.9, 35.4)	
Supports child activity at the weekend	Mother (ref)	0	0	0	0.33
	Father	13.1 (-26.5, 52.6)	55.6 (-7.2, 118.3)	-26.2 (-75.9, 23.4)	
	Both parents	22.6 (-7.7, 52.9)	52.8 (1.8, 103.7)	4.7 (-31.3, 40.7)	

For peer review only

For peer review only - http://bmjopen.bmj.com/site/about/guidelines.xhtml

MVPA: Moderate-to-vigorous physical activity; Models are adjusted for child age, parent gender and household IMD score

Table 3 Odds ratio for children achieving 60 minutes of MVPA per day associated with gender of parent supporting child physical activity during the week and weekend (N=944)

**Exposure**		**Meeting government guideline: odds ratio (95% confidence interval)**			**P for gender interaction**
		**All (N=944)**	**Boys (N=427)**	**Girls (N=517)**	
**Supports child activity during week**	Mother (ref)	0	0	0	0.95
	Father	0.96 (0.54, 1.72)	1.61 (0.62, 4.21)	0.75 (0.34, 1.66)	
	Both parents	1.60 (1.20, 2.14)	2.23 (1.37, 3.62)	1.23 (0.83, 1.82)	
**Supports child activity at the weekend**	Mother (ref)	0	0	0	0.30
	Father	1.20 (0.78, 1.86)	2.10 (1.02, 4.32)	0.74 (0.40, 1.38)	
	Both parents	1.20 (0.86, 1.68)	1.81 (1.01, 3.24)	1.00 (0.64, 1.54)	

For peer review only

For peer review only - http://bmjopen.bmj.com/site/about/guidelines.xhtml

MVPA: Moderate-to-vigorous physical activity; Models are adjusted for child age, parent gender and household IMD score

For peer review only

For peer review only - http://bmjopen.bmj.com/site/about/guidelines.xhtml

DISCUSSION

The data presented in this paper show that while the participants in this study believe the responsibility of supporting child physical activity should be shared between both parents, quantitative data suggest that families mostly share the role on the weekend, with mothers primarily supporting child activity during the week. This finding was mirrored in the interview data, where several mothers reported that they supported child activity during the week, because fathers worked long hours or late into the evening. Despite families traditionally functioning such that one parent (often the mother) takes on more childcare responsibilities in general, it is interesting that parents still feel that supporting child activity should be a shared responsibility. Indeed, traditional familial roles are shifting, and it is now more common for both parents to work and for fathers to take on the role of primary care provider,[54 55] so it may be expected that more fathers are taking an active role in their children's physical activity. We found that the majority of parents reported they shared the role of supporting their child's activity both during the week and at the weekend (40-65% of mothers and fathers responded this way for both time points; Table S2).

In quantitative analyses for all three outcomes (time spent in MVPA, meeting MVPA recommendations and CPM) we saw similar patterns of, in general, higher child physical activity where parents reportedly shared the role of supporting their child's physical activity during both weekdays and weekends. For example, both parents supporting child activity equally during the week was associated with boys doing an additional 40 minutes of MVPA across the week, which could be the difference between a child achieving the recommended guidelines or not. The one exception was for meeting MVPA recommendations at the weekend, where associations of fathers reportedly leading the support were similar to those

For peer review only - http://bmjopen.bmj.com/site/about/guidelines.xhtml

when both parents shared the responsibility. There was some evidence that positive associations were stronger for sons, and that some associations were inverse for daughters. However, we found no strong statistical evidence that associations differed between sons and daughters, and without further exploration in much larger numbers we cannot assume that parental roles in supporting their child's activity differ by the child's gender.

There was some suggestion that mothers were more likely to support their daughter to be active, while fathers were more likely to support their son's activity, though caution is needed here given the disparity in which parents provide data, with 72% of families having data from mothers only and 28% from fathers only. Several studies have reported that fathers may be more involved in their son's physical activity,[15 31] or have found stronger links between father-son and mother-daughter dyads in terms of their physical activity behaviour.[36-38] In contrast, interview data from the current study revealed a myriad of gender patterns, including examples from fathers supporting girls' physical activity because they were more confident than mothers in supporting physical activity or because they enjoy watching their daughter play football, and a mother taking her son mountain biking to engage in quality one-on-one time. There were also examples of fathers taking sons to traditionally male-orientated sports (e.g., rugby or football) to bond over shared interests and give mothers a respite from parenting.

The results from the current study suggest intervention studies should be developed to engage both parents, or specifically fathers, in supporting their children to be active, not necessarily focused on children and parents being active together, but rather on how parents can work together to schedule times for children to be active across the week in both structured and

For peer review only - http://bmjopen.bmj.com/site/about/guidelines.xhtml

unstructured activities, and how parents can share the role between parenting partners. Table 4 summarises the key findings and implications for how parents can support child activity that have emerged from this study. These suggestions provide ways that researchers and policy makers can help parents to support their child's physical activity, through providing advice and encouragement to developing family physical activity plans. Research needs to be conducted into how best to operationalise these suggestions and understand the channels that parents typically use for finding parenting advice and ideas for physical activities. Potential avenues for disseminating advice include encouraging sharing of advice and positive affirmations via parents' peer networks, delivering information through schools, or communicating advice via social media and parenting forums.

For peer review only

For peer review only - http://bmjopen.bmj.com/site/about/guidelines.xhtml

Table 4 Key findings and implications for how parents can support their child's physical activity

Finding	Implication
Mothers primarily support child physical activity during the week	Develop advice for mothers to help them facilitate their child's physical activity during busy weekdays (e.g., identifying times in the day for promoting activity, ideas for active games)
Engaging fathers to be involved in supporting child physical activity is important	Encourage fathers to see the important role they can play in supporting their child's activity
Children, possibly more so boys, are more active if both parents share the role of supporting child physical activity	Develop family physical activity plans (e.g., who can support when) to encourage both parents to take an active role in supporting their child's physical activity
Parents can use physical activity time to bond over shared interests or engage in quality one-to-one time with children	Encourage parents to value physical activity time as a way to share interests and bond with children (e.g., promote physical activity as quality family time)
Some parents, possibly more so mothers, struggle for confidence when it comes to supporting child physical activity	Develop parental skills and confidence in supporting and facilitating child activity, and encourage parents to model the behaviours that they wish their child to adopt

For peer review only

Strengths and limitations

A main strength of the study is the mixed-methods approach, utilising both accelerometer-assessed physical activity from a large sample of 8-9-year-old children and semi-structured interview data with parents. This approach provides rich data about the gender roles associated with how parents support their child's activity. Another strength is that we interviewed a relatively large sample of parents, including 20 fathers, a group that are known to be difficult to engage in research.[56] Limitations of the study include its cross-sectional nature so causality could not be examined. In the main dataset, parents were primarily represented by mothers (72%), which is likely to have biased how they responded to questions about who supports their child's activity. In addition, because only one parent was required to participate with their child, this study does not include information on whether children were from same-sex families, single-parent families, or where primary caregivers are grandparent or extended family. We had very limited power to explore gender interactions, thus whilst our results suggest that parent support of their child's physical activity might have a stronger positive impact on sons compared with daughters it would be wrong to conclude that from these data, and much larger independent studies are required to explore that further. Parental responses to our exposure questions provided no information on the type (quality or quantity) of their supporting role, and thus it is not known whether both parents equally supporting child activity is simply a proxy for greater support. Additionally, the variable ascertaining which parent 'should take the lead in supporting child physical activity' did not differentiate between weekdays and weekend days. 279 families were excluded from the study due to missing data, which may have resulted in sampling bias, because these participants differed from included participants in terms of their MVPA and household IMD score. This study is also drawn from a single UK city area with a primarily White British

For peer review only - http://bmjopen.bmj.com/site/about/guidelines.xhtml

population, and as such our ability to extend findings to other settings countries, and ethnicities is limited.

CONCLUSIONS

We found some evidence that parents share the role of supporting their children to be active. It is possible that mothers primarily support child activity during the week, with the role shared more equally on the weekend. Children are more active when parents share the responsibility of supporting their child's activity, but further large independent studies are required to replicate our findings and determine whether parental support has a stronger effect on sons than daughters. Future studies should also seek to engage more fathers, verify reports of who takes a supporting role (for example through cross comparison of reports from each parent and the child or direct observation), and to collect information on the nature of supporting roles (quality and frequency).

ACKNOWLEDGEMENTS

This work was supported by grants from the British Heart Foundation (ref PG/11/51/28986 and SP 14/4/31123). DAL works in a unit that receives funding from the University of Bristol and UK Medical Research Council (MC_UU_1201/5); she is also a UK National Institute of Health and Research Senior Investigator (NF-SI-0166-10196). The funders had no involvement in data analysis, data interpretation or writing of the paper.

We would like to thank all of the families and schools that have taken part in the B-PROACT1V project. We would also like to thank all current and previous members of the research team who are not authors on this paper.

For peer review only - http://bmjopen.bmj.com/site/about/guidelines.xhtml

COMPETING INTERESTS

All authors have completed the ICMJE uniform disclosure form at www.icmje.org/coi_disclosure.pdf and declare: all authors had financial support from the British Heart Foundation for the submitted work; no financial relationships with any organisations that might have an interest in the submitted work in the previous three years; no other relationships or activities that could appear to have influenced the submitted work.

CONTRIBUTORS

Conception / design**:** RJ, ESM, JLT, DAL and SJS.

Quantitative and Qualitative data collection: ESM.

Data analysis / acquisition/ interpretation: ESM, RJ, ZT and DAL.

Drafting / revising critically for important content: All authors.

Final approval: All authors.

Accountability for study and manuscript: ESM, RJ.

DATA SHARING STATEMENT

The datasets generated during the current study are not publicly available as the project is ongoing and data are not ready for archiving. We will make quantitative data available to the wider research community once the project is complete in August 2019. Because of possible disclosure with qualitative data we will consider requests to use and further explore those data on a per request basis with an appropriate balance between sharing data as fully as possible whilst maintaining participant anonymity.

REFERENCES

For peer review

1. Janssen I, LeBlanc AG. Systematic review of the health benefits of physical activity and fitness in school-aged children and youth. Int J Behav Nutr Phys Act 2010;**7**:40 doi: 10.1186/1479-5868-7-40.
2. Strong WB, Malina RM, Blimkie CJ, et al. Evidence based physical activity for school-age youth. J Pediatr 2005;**146**(6):732-7
3. Department of Health, Physical Activity, Health Improvement and Protection. Start Active, Stay Active: A report on physical activity from the four home countries' Chief Medical Officers. London, 2011.
4. Griffiths LJ, Cortina-Borja M, Sera F et al. How active are our children? Findings from the Millennium Cohort Study. BMJ Open 2013;**3**(8):e002893 doi: 10.1136/bmjopen-2013-002893.
5. Scholes S. Health Survey for England 2015: Physical activity in children. Health and Social Care Information Centre. London, 2016.
6. Cooper AR, Goodman A, Page AS, et al. Objectively measured physical activity and sedentary time in youth: the International children's accelerometry database (ICAD). Int J Behav Nutr Phys Act 2015;**12**:113 doi: 10.1186/s12966-015-0274-5.
7. Nader PR, Bradley RH, Houts RM, McRitchie SL, O'Brien M. Moderate-to-vigorous physical activity from ages 9 to 15 years. JAMA 2008;**300**(3):295-305.
8. Farooq MA, Parkinson KN, Adamson AJ, et al. Timing of the decline in physical activity in childhood and adolescence: Gateshead Millennium Cohort Study. Br J Sports Med 2017:1-6 doi:10.1136/bjsports-2016-096933.
9. Jago R, Solomon-Moore E, Macdonald-Wallis C, Sebire SJ, Thompson JL, Lawlor DA. Change in children's physical activity and sedentary time between Year 1 and Year 4 of primary school in the B-PROACT1V cohort. Int J Behav Nutr Phys Act 2017;**14**:33 doi: 10.1186/s12966-017-0492-0.

For peer review only - http://bmjopen.bmj.com/site/about/guidelines.xhtml

10. Patrick H, Hennessy E, McSpadden K, Oh A. Parenting styles and practices in children's obesogenic behaviors: scientific gaps and future research directions. Child Obes 2013;**9**(S1):S73-86 doi: 10.1089/chi.2013.0039.

11. Davison KK, Masse LC, Timperio A, et al. Physical activity parenting measurement and research: challenges, explanations, and solutions. Child Obes 2013;**9**(Suppl):S103-9 doi: 10.1089/chi.2013.0037.

12. Sleddens EF, Gerards SM, Thijs C, de Vries NK, Kremers SP. General parenting, childhood overweight and obesity-inducing behaviors: a review. Int J Pediatr Obes 2011;**6**(2-2):e12-27 doi: 10.3109/17477166.2011.566339.

13. O'Connor TM, Jago R, Baranowski T. Engaging parents to increase youth physical activity: a systematic review. Am J Prev Med 2009;**37**(2):141-9 doi: 10.1016/j.amepre.2009.04.020.

14. Jago R, Fox KR, Page AS, Brockman R, Thompson JL. Parent and child physical activity and sedentary time: do active parents foster active children? BMC Public Health 2010;**10**(1):194 doi: 10.1186/1471-2458-10-194.

15. Gustafson SL, Rhodes RE. Parental correlates of physical activity in children and early adolescents. Sports Med 2006;**36**(1):79-97.

16. Edwardson CL, Gorely T. Parental influences on different types and intensities of physical activity in youth: A systematic review. Psychol Sport Exerc 2010;**11**(6):522-35 doi: 10.1016/j.psychsport.2010.05.001.

17. Jago R, Sebire SJ, Wood L, et al. Associations between objectively assessed child and parental physical activity: a cross-sectional study of families with 5-6 year old children. BMC Public Health 2014;**14**:655 doi: 10.1186/1471-2458-14-655.

18. Jago R, Solomon-Moore E, Macdonald-Wallis C, Thompson JL, Lawlor DA, Sebire SJ. Association of parents' and children's physical activity and sedentary time in

For peer review only - http://bmjopen.bmj.com/site/about/guidelines.xhtml

Year 4 (8-9) and change between Year 1 (5-6) and Year 4: a longitudinal study. Int J Behav Nutr Phys Act 2017;**14**:110 doi: 10.1186/s12966-017-0565-0.

19. Fuemmeler BF, Anderson CB, Mâsse LC. Parent-child relationship of directly measured physical activity. Int J Behav Nutr Phys Act 2011;8:17 doi: 10.1186/1479-5868-8-17.

20. Garriguet D, Colley R, Bushnik T. Parent-Child association in physical activity and sedentary behaviour. Health Reports 2017;**28**(6):3-11.

21. Erkelenz N, Kobel S, Kettner S, et al. Parental activity as influence on children's BMI percentiles and physical activity. J Sports Sci Med 2014;**13**:645-50.

22. Tate EB, Shah A, Jones M, et al. Toward a better understanding of the link between parent and child physical activity levels: The moderating role of parental encouragement. J Phys Act Health 2015;**12**:1238-44 doi: 10.1123/jpah.2014-0126.

23. Hennessy E, Hughes SO, Goldberg JP, et al. Parent-child interactions and objectively-measured child physical activity: a cross-sectional study. Int J Behav Nutr Phys Act 2010;**7**:71 doi: 10.1186/1479-5868-7-71.

24. Tilly L, Scott J. Women, Work and Family. New York: Holt, Reinhart and Winston; 1978.

25. Goldscheider F, Bernhardt E, Lappegard T. The Gender Revolution: A Framework for Understanding Changing Family and Demographic Behavior. Popul Dev Rev 2015;**41**(2):207-239.

26. Gerson K. The Unfinished Revolution: Coming of Age in a New Era of Gender, Work, and Family. New York: Oxford University Press; 2010.

27. Hofferth S, Pleck J, Goldscheider F, Curtin S, Hrapezynski K. Changing family structure and men's motivation for parenthood and parenting in the U.S. In:

For peer review only

For peer review only - http://bmjopen.bmj.com/site/about/guidelines.xhtml

Handbook of Father Involvement: Multidisciplinary Perspectives. 2nd ed. Cabrera NJ, Tamis-LeMonda CS, editors. Taylor and Francis; 2012. p. 57-80.

28. Davison KK, Cutting TM, Birch LL. Parents' activity-related parenting practices predict girls' physical activity. Med Sci Sports Exerc 2003;**35**:1589-95 doi: 10.1249/01.MSS.0000084524.19408.0C.

29. Lloyd AB, Lubans DR, Plotnikoff RC, Collins CE, Morgan PJ. Maternal and paternal parenting practices and their influence on children's adiposity, screen-time, diet and physical activity. Appetite 2014;**79**:149-57 doi: 10.1016/j.appet.2014.04.010.

30. Davison KK, Gicevic S, Aftosmes-Tobio A, et al. Fathers' representation in observational studies on parenting and childhood obesity: a systematic review and content analysis. Am J Public Health 2016;**106**(11):e14-e21 doi: 10.2105/AJPH.2016.303391.

31. Zahra J, Sebire SJ, Jago R. "He's probably more Mr. sport than me" – a qualitative exploration of mothers' perceptions of fathers' role in their children's physical activity. BMC Pediatrics 2015;15:101 doi: 10.1186/s12887-015-0421-9.

32. Vollmer RL, Adamsons K, Gorin A, Foster JS, Mobley AR. Investigating the relationship of body mass index, diet quality, and physical activity level between fathers and their preschool-aged children. J Acad Nutr Diet 2015;115:919-26 doi: 10.1016/j.jand.2014.12.003.

33. Schoeppe S, Liersch S, Röbl M, Krauth C, Walter U. Mothers and fathers both matter: the positive influence of parental physical activity modelling on children's leisure-time physical activity. Pediatr Exerc Sci 2016;**28**:466-72 doi: 10.1123/pes.2015-0236.

For peer review only - http://bmjopen.bmj.com/site/about/guidelines.xhtml

34. Morgan PJ, Collins CE, Plotnikoff RC, et al. The ‘healthy dads, healthy kids’ community randomized controlled trial: a community-based healthy lifestyle program for fathers and their children. Prev Med 2014;**61**:90-99 doi: 10.1016/j.ypmed.2013.12.019.

35. Morgan PJ, Lubans DR, Callister R, et al. The ‘healthy dads, healthy kids’ randomized controlled trial: efficacy of a healthy lifestyle program for overweight fathers and their children. Int J Obes 2011;**35**(3):436-47 doi: 10.1038/ijo.2010.151.

36. Eriksson M, Nordqvist T, Rasmussen F. Associations between parents’ and 12-year-old children’s sport and vigorous activity: the role of self-esteem and athletic competence. J Phys Act Health 2008;5(3):359-73 doi: 10.1123/jpah.5.3.359.

37. O’Loughlin J, Paradis G, Kishchuk N, Barnett T, Renaud L. Prevalence and correlates of physical activity behaviors among elementary schoolchildren in multi-ethnic, low income, inner-city neighborhoods in Montreal, Canada. Ann Epidemiol 1999;**9**(7):397-407.

38. Trost SG, Pate RR, Saunders R, Ward DS, Dowda M, Felton G. A prospective study of the determinants of physical activity in rural fifth-grade children. Prev Med 1997;**26**(2):257-63.

39. Trost SG, Pate RR, Ward DS, Saunders R, Riner W. Determinants of physical activity in active and low-active, sixth grade African-American youth. J Sch Health 1999;**69**(1):29-34.

40. Jago R, Thompson JL, Sebire SJ, et al. Cross-sectional associations between the screen-time of parents and young children: differences by parent and child gender and day of the week. Int J Behav Nutr Phys Act 2014;**11**:54 doi: 10.1186/1479-5868-11-54.

For peer review only - http://bmjopen.bmj.com/site/about/guidelines.xhtml

41. Creswell, J. Research design: Qualitative, quantitative, and mixed methods approaches. Thousand Oaks, CA: Sage, 1997.

42. Jago R, Bailey R. Ethics and paediatric exercise science: Issues and making a submission to a local ethics and research committee. J Sports Sci 2001;**19**(7):527-535.

43. Payau MR, Adolph AL, Vohra FA, Butte NF. Validation and calibration of physical activity monitors in children. Obes Res 2002;**10**(3):150-7. doi: 10.1038/oby.2002.24

44. Pate RR, Almeida MJ, McIver KL, Pfeiffer KA, Dowda M. Validation and Calibration of an Accelerometer in Preschool Children. Obes 2006;**14**(11):2000-2006. doi: 10.1038/oby.2006.234

45. Evenson KR, Catellier DJ, Gill K, et al. Calibration of two objective measures of physical activity for children. J Sport Sci 2008;**26**:1557-65 doi: 10.1080/02640410802334196.

46. Trost SG, Loprinzi PD, Moore R, Pfeiffer KA. Comparison of accelerometer cut points for predicting activity intensity in youth. Med Sci Sports Exerc 2011;**43**(7):1360-1368. doi: 10.1249/MSS.0b013e318206476e.

47. Department for Communities and Local Government. The English Indices of Deprivation 2015 Statistical Release. Office for National Statistics 2015.

48. Kesten J, Jago R, Sebire SJ, et al. Understanding the accuracy of parental perceptions of child physical activity: a mixed methods analysis. J Phys Act Health 2015;**12**(12):1529–35. doi: 10.1123/jpah.2014-0442.

49. Jago R, Zahra J, Edwards MJ, et al. Managing the screen-viewing behaviours of 5-6 year old children: a qualitative analysis of parental strategies. BMJ Open 2016;**6**(3): e010355. doi: 10.1136/bmjopen-2015-010355.

For peer review only - http://bmjopen.bmj.com/site/about/guidelines.xhtml

50. Thompson JL, Sebire SJ, Kesten JM, et al. How parents perceive screen viewing in their 5-6 year old child within the context of their own screen viewing time: a mixed-methods study. BMC Public Health 2017;**17**:471. doi: 10.1186/s12889-017-4394-5.

51. Sebire SJ, Jago R, Wood L, et al. Examining a conceptual model of parental nurturance, parenting practices and physical activity among 5-6 year olds. Soc Sci Med 2016;**148**:18-24. doi: 10.1016/j.socscimed.2015.11.022.

52. Elo S, Kyngäs H. The qualitative content analysis process. J Adv Nurs 2008;**62**(1): 107-15 doi: 10.1111/j.1365-2648.2007.04569.x.

53. Gale NK, Heath G, Cameron E, Rashid S, Redwood S. Using the framework method for the analysis of qualitative data in multi-disciplinary health research. BMC Med Res Methodol 2013;**13**:117 doi: 10.1186/1471-2288-13-117.

54. Anderson PM, Butcher KF. Childhood obesity trends and potential causes. Future Child 2006;**16**:19-45 doi: 10.1353/foc.2006.0001.

55. Laughlin L. Who's minding the kids? Child care arrangements: spring 2011. https://www.census.gov/prod/2013pubs/p70-135.pdf. Published April 2013.

56. Macfadyen A, Swallow V, Santacroce S, Lambert H. Involving fathers in research. J Spec Pediatr Nurs 2011;**16**(3):216-9 doi: 10.1111/j.1744-6155.2011.00287.x

Figure 1 Study flow of participants for the quantitative study

Figure 2 Study flow of participants for the qualitative study

For peer review only - http://bmjopen.bmj.com/site/about/guidelines.xhtml

Figure 1 Study flow of participants for the quantitative study

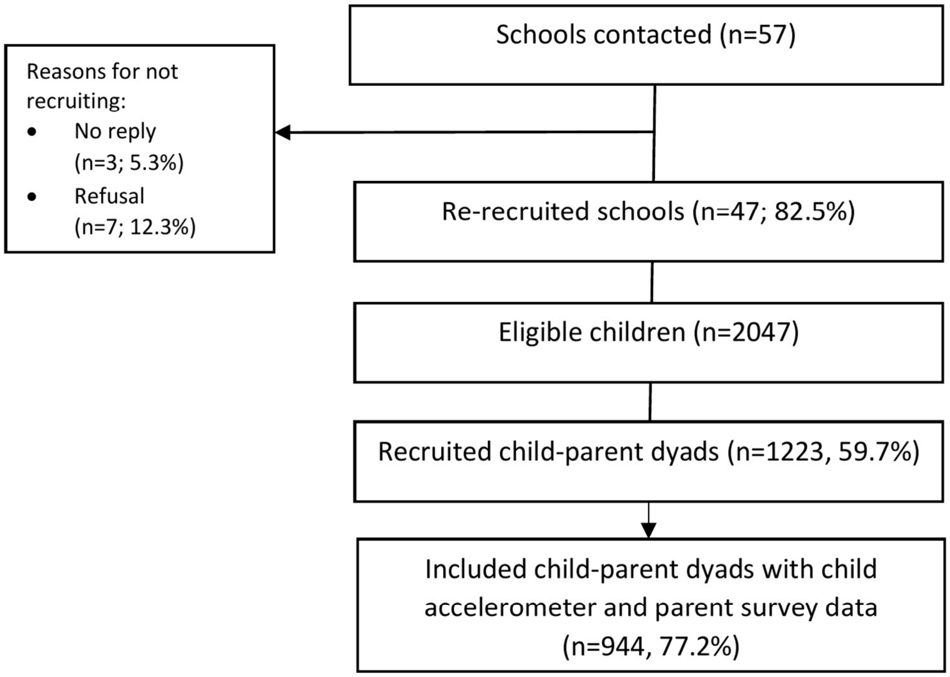

Figure 1 Study flow of participants for the quantitative study

132x104mm (300 x 300 DPI)

For peer review only - http://bmjopen.bmj.com/site/about/guidelines.xhtml

Figure 2 Study flow of participants for the qualitative study

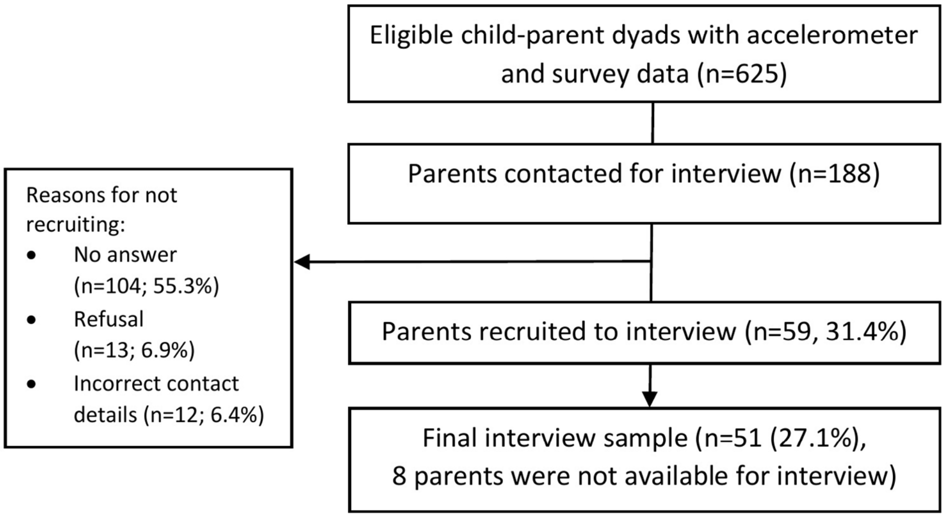

Figure 2 Study flow of participants for the qualitative study

132x81mm (300 x 300 DPI)

review only

Table S1 Number of mothers and fathers who were eligible for and recruited to the interview study

No.	Sub-group description	N parents eligible in each sub-group Total	Mothers	Fathers	N parents recruited in each sub-group Total	Mothers	Fathers
1	Low MVPA, low SED boys	31	20	11	6	4	2
2	High MVPA, low SED boys	116	82	34	6	3	3
3	Low MVPA, high SED boys	67	51	16	7	5	2
4	High MVPA, high SED boys	63	48	15	6	3	3
5	Low MVPA, low SED girls	69	48	21	6	3	3
6	High MVPA, low SED girls	86	67	19	6	4	2
7	Low MVPA, high SED girls	138	111	27	7	4	3
8	High MVPA, high SED girls	55	37	18	7	5	2
	**Total**	**625**	**464**	**161**	**51**	**31**	**20**

For peer review only

Table S2 Frequency of reporting which parent supports child activity by parent and separately by child gender

		Parent gender			Child gender		
		Males (N=264) %	Females (N=680) %	Chi-squared p-value for difference	Boys (N=427) %	Girls (N=517) %	Chi-squared p-value for difference
**Supports child activity during the week**	Mother	26.9	57.4	<0.001	44.5	52.4	0.04
	Father	16.7	2.9		6.6	7.0	
	Both parents	56.4	39.7		48.9	40.6	
**Supports child activity at the weekend**	Mother	8.3	30.7	<0.001	21.1	27.3	0.02
	Father	26.9	14.1		20.8	15.1	
	Both parents	64.8	55.1		58.1	57.6	
**Who should support child activity**	Mother	1.1	6.9	0.001	4.2	6.0	0.07
	Father	1.5	0.7		1.6	0.4	
	Both parents	97.3	92.4		94.1	93.6	

For peer review only

Who leads and who supports? A cross-sectional mixed methods study of mothers' and fathers' support for child physical activity

STROBE Statement—checklist of items that should be included in reports of observational studies

	Item No	Recommendation
**Title and abstract**	1	(*a*) Indicate the study's design with a commonly used term in the title or the abstract Title Page
		(*b*) Provide in the abstract an informative and balanced summary of what was done and what was found – Pages 2-3
**Introduction**		
Background/rationale	2	Explain the scientific background and rationale for the investigation being reported – Pages 5-6
Objectives	3	State specific objectives, including any prespecified hypotheses – Page 6.
**Methods**		
Study design	4	Present key elements of study design early in the paper Pages 6-7.
Setting	5	Describe the setting, locations, and relevant dates, including periods of recruitment, exposure, follow-up, and data collection Pages 6-7.
Participants	6	(*a*) *Cohort study*—Give the eligibility criteria, and the sources and methods of selection of participants. Describe methods of follow-up<br>*Case-control study*—Give the eligibility criteria, and the sources and methods of case ascertainment and control selection. Give the rationale for the choice of cases and controls<br>*Cross-sectional study*—Give the eligibility criteria, and the sources and methods of selection of participants Page 7
		(*b*) *Cohort study*—For matched studies, give matching criteria and number of exposed and unexposed<br>*Case-control study*—For matched studies, give matching criteria and the number of controls per case
Variables	7	Clearly define all outcomes, exposures, predictors, potential confounders, and effect modifiers. Give diagnostic criteria, if applicable - Pages 7-9
Data sources/ measurement	8*	For each variable of interest, give sources of data and details of methods of assessment (measurement). Describe comparability of assessment methods if there is more than one group Pages 7-9
Bias	9	Describe any efforts to address potential sources of bias – Page 8
Study size	10	Explain how the study size was arrived at - Pages 7-8 (Figures 1 & 2)
Quantitative variables	11	Explain how quantitative variables were handled in the analyses. If applicable, describe which groupings were chosen and why - Pages 10-11
Statistical methods	12	(*a*) Describe all statistical methods, including those used to control for confounding – Pages 10-11
		(*b*) Describe any methods used to examine subgroups and interactions – Page 11
		(*c*) Explain how missing data were addressed - Pages 10-11
		(*d*) *Cohort study*—If applicable, explain how loss to follow-up was addressed<br>*Case-control study*—If applicable, explain how matching of cases and controls was

For peer review only - http://bmjopen.bmj.com/site/about/guidelines.xhtml

		addressed<br>*Cross-sectional study*—If applicable, describe analytical methods taking account of sampling strategy - Pages 10-11
		(*e*) Describe any sensitivity analyses – Page 11

Continued on next page

For peer review only

For peer review only - http://bmjopen.bmj.com/site/about/guidelines.xhtml

**Results**		
Participants	13*	(a) Report numbers of individuals at each stage of study—eg numbers potentially eligible, examined for eligibility, confirmed eligible, included in the study, completing follow-up, and analysed Table 1 (Figure 1 & 2)
		(b) Give reasons for non-participation at each stage - Figure 1 & 2
		(c) Consider use of a flow diagram – Figure 1 & 2
Descriptive data	14*	(a) Give characteristics of study participants (eg demographic, clinical, social) and information on exposures and potential confounders – Table 1
		(b) Indicate number of participants with missing data for each variable of interest - Table 1
		(c) *Cohort study*—Summarise follow-up time (eg, average and total amount)
Outcome data	15*	*Cohort study*—Report numbers of outcome events or summary measures over time
		Case-control study—Report numbers in each exposure category, or summary measures of exposure
		Cross-sectional study—Report numbers of outcome events or summary measures Table 1
Main results	16	(*a*) Give unadjusted estimates and, if applicable, confounder-adjusted estimates and their precision (eg, 95% confidence interval). Make clear which confounders were adjusted for and why they were included – Tables 2-3
		(*b*) Report category boundaries when continuous variables were categorized - Tables 2-3
		(*c*) If relevant, consider translating estimates of relative risk into absolute risk for a meaningful time period Discussion – N/A
Other analyses	17	Report other analyses done—eg analyses of subgroups and interactions, and sensitivity analyses – Supplementary tables
**Discussion**		
Key results	18	Summarise key results with reference to study objectives – Page 22
Limitations	19	Discuss limitations of the study, taking into account sources of potential bias or imprecision. Discuss both direction and magnitude of any potential bias - Page 25
Interpretation	20	Give a cautious overall interpretation of results considering objectives, limitations, multiplicity of analyses, results from similar studies, and other relevant evidence – Page 25-26
Generalisability	21	Discuss the generalisability (external validity) of the study results Page 25
**Other information**		
Funding	22	Give the source of funding and the role of the funders for the present study and, if applicable, for the original study on which the present article is based – Page 26

*Give information separately for cases and controls in case-control studies and, if applicable, for exposed and unexposed groups in cohort and cross-sectional studies.

Note: An Explanation and Elaboration article discusses each checklist item and gives methodological background and published examples of transparent reporting. The STROBE checklist is best used in conjunction with this article (freely available on the Web sites of PLoS Medicine at http://www.plosmedicine.org/, Annals of Internal Medicine at http://www.annals.org/, and Epidemiology at http://www.epidem.com/). Information on the STROBE Initiative is available at www.strobe-statement.org.

For peer review only

For peer review only - http://bmjopen.bmj.com/site/about/guidelines.xhtml

COREQ (COnsolidated criteria for REporting Qualitative research) Checklist

A checklist of items that should be included in reports of qualitative research. You must report the page number in your manuscript where you consider each of the items listed in this checklist. If you have not included this information, either revise your manuscript accordingly before submitting or note N/A.

Topic	Item No.	Guide Questions/Description	Reported on Page No.
**Domain 1: Research team and reflexivity**			
Personal characteristics			
Interviewer/facilitator	1	Which author/s conducted the interview or focus group?	Page 27
Credentials	2	What were the researcher's credentials? E.g. PhD, MD	Page 9
Occupation	3	What was their occupation at the time of the study?	Page 9
Gender	4	Was the researcher male or female?	Page 9
Experience and training	5	What experience or training did the researcher have?	Page 9
Relationship with participants			
Relationship established	6	Was a relationship established prior to study commencement?	Pages 8-9
Participant knowledge of the interviewer	7	What did the participants know about the researcher? e.g. personal goals, reasons for doing the research	N/A
Interviewer characteristics	8	What characteristics were reported about the inter viewer/facilitator? e.g. Bias, assumptions, reasons and interests in the research topic	N/A
**Domain 2: Study design**			
Theoretical framework			
Methodological orientation and Theory	9	What methodological orientation was stated to underpin the study? e.g. grounded theory, discourse analysis, ethnography, phenomenology, content analysis	Page 11
Participant selection			
Sampling	10	How were participants selected? e.g. purposive, convenience, consecutive, snowball	Pages 8-9
Method of approach	11	How were participants approached? e.g. face-to-face, telephone, mail, email	Page 9
Sample size	12	How many participants were in the study?	Page 9
Non-participation	13	How many people refused to participate or dropped out? Reasons?	Page 9 (Fig 2)
Setting			
Setting of data collection	14	Where was the data collected? e.g. home, clinic, workplace	Page 8
Presence of non-participants	15	Was anyone else present besides the participants and researchers?	N/A
Description of sample	16	What are the important characteristics of the sample? e.g. demographic data, date	Pages 8-9, 12 (
Data collection			
Interview guide	17	Were questions, prompts, guides provided by the authors? Was it pilot tested?	Page 9
Repeat interviews	18	Were repeat inter views carried out? If yes, how many?	N/A
Audio/visual recording	19	Did the research use audio or visual recording to collect the data?	Page 9
Field notes	20	Were field notes made during and/or after the inter view or focus group?	N/A
Duration	21	What was the duration of the inter views or focus group?	Page 12
Data saturation	22	Was data saturation discussed?	Page 9
Transcripts returned	23	Were transcripts returned to participants for comment and/or	N/A

For peer review only

Topic	Item No.	Guide Questions/Description	Reported on Page No.
		correction?	
**Domain 3: analysis and findings**			
Data analysis			
Number of data coders	24	How many data coders coded the data?	Page 11
Description of the coding tree	25	Did authors provide a description of the coding tree?	N/A
Derivation of themes	26	Were themes identified in advance or derived from the data?	Page 11
Software	27	What software, if applicable, was used to manage the data?	Page 11
Participant checking	28	Did participants provide feedback on the findings?	N/A
Reporting			
Quotations presented	29	Were participant quotations presented to illustrate the themes/findings? Was each quotation identified? e.g. participant number	Pages 14-17
Data and findings consistent	30	Was there consistency between the data presented and the findings?	Pages 14-17
Clarity of major themes	31	Were major themes clearly presented in the findings?	Pages 14-17
Clarity of minor themes	32	Is there a description of diverse cases or discussion of minor themes?	Pages 14-17

Developed from: Tong A, Sainsbury P, Craig J. Consolidated criteria for reporting qualitative research (COREQ): a 32-item checklist for interviews and focus groups. *International Journal for Quality in Health Care*. 2007. Volume 19, Number 6: pp. 349 – 357

Once you have completed this checklist, please save a copy and upload it as part of your submission. DO NOT include this checklist as part of the main manuscript document. It must be uploaded as a separate file.

For peer review only
